# Unlocking the African bioeconomy and strengthening biodiversity conservation through genomics and bioinformatics

**DOI:** 10.1038/s44185-025-00102-9

**Published:** 2025-07-29

**Authors:** Ichrak Hayah, Victor Ezebuiro, Samuel Paul Kagame, Josiah Ochieng Kuja, Cecilia Waruhiu, Lucky Tendani Nesengani, Sinebongo Mdyogolo, Annelin Henriehetta Molotsi, Priscilla Abechi, Asmaa Mohamed Abushady, Nabil Amor, Brian Andika, Abdelhamid Barakat, Girish Beedessee, Marietjie Botes, Xavier David, Nzube Ebuzoeme, Zewdu Edea, Achraf El Allali, Owanate Pearl Elekima, Assem Kadry Elsherif, Semir Bechir Suheil Gaouar, Yohannes Gedamu Gebre, Owunari Abraham Georgewill, Lydia Hadjeras, Mohammed Ahmed Hassan, Mohamed Hijri, Isidore Houaga, Justin Eze Ideozu, Matthias Igoh, Mary Paschal Iwundu, Syed Arif Sulthan Jaffer Ali, Atef Jaouani, Ahmed Marwane Kermouni Serradj, Radjaa Khedim, Mariëtte Kilian, Dennis Manthi Kivuva, Mehdi Knidiri, Komi Komi Koukoura, Eleojo Roseline Kwasi, Kim Labuschagne, Antoine Lusala Mafwila, Isabel Mensah, Uzoma Modebelu, Prudent Mokgokong, Morad M. Mokhtar, Sadik Muzemil, Helen Nigussie, Valentine Otang Ntui, Joel Ogwang, Nicholas Abraham Olivier, Olanrewaju Olufowobi, Taiwo Crossby Omotoriogun, Onikepe Folarin, Philomena Eromon, Jeremiah Orina, Faissal Ouardi, Tracy Parish, Mercy Peter, Jacques Potgieter, Fouzia Radouani, Madeleine Ramantswana, Shaimaa Roshdy Abdullah Reda, Samson Pandam Salifu, Sarah Bingaman Schwartz, Ntji Shabangu, Abdoallah Sharaf, Iyeopu Minakiri Siminialayi, Rae Marvin Smith, Hiroaki Taniguchi, Preye Maureen Tari-Ukuta, Kassahun Tesfaye, Fatim Zohra Tmimi, Libert Brice Tonfack, Ogbuagu Ugorji Udensi, Victoria Wavinya Wambua, Sammy Wambua, Kennedy Were, Timipanipiri ThankGod Wood, Bret Mark Wurdeman, Yedomon Ange Bovys Zoclanclounon, Andrews Frimpong Adu, Sotonye Leslie Gillis-Harry, Nicholas Kwasi-Do Ohene Opoku, Thendo Stanley Tshilate, Siyeofori Dede, Soala Obie Minimah, Yves Hermandez Tchiechoua, Andreas Gisel, Chadlia Hamdi, Tshepo Mafokwane, Blessing Adanta Odogwu, Gift Nwachukwu, Zahra Mungloo-Dilmohamud, Faten Ghodhbane-Gtari, Chinagorom Ibeachu, Renate Dorothea Zipfel, Wenceslaus C. Madu, Johnpaul Chukwudi Okorocha, Tracy Masebe, Kilsi Borgbara, Wynand Goosen, Suereta Fortuin, Kristien Nel Van Zyl, Ongeziwe Mbhele, Anise Happi, Christian Happi, Ntanganedzeni Mapholi, Julian Onyewuonyeoma Osuji, Anne WT Muigai, ThankGod Echezona Ebenezer, Bouabid Badaoui

**Affiliations:** 1 Faculty of Sciences, Mohammed V University in Rabat, Rabat, Morocco; 2 African Genome Center, University Mohammed VI Polytechnic (UM6P), Ben Guerir, Morocco; 3 Regional Centre for Biotechnology and Bioresources Research & South-South Zonal Centre of Excellence, University of Port Harcourt, Port Harcourt, Nigeria; 4 Ghent University, Ghent, Belgium; 5 Washington State University, Global Health Kenya, Nairobi, Kenya; 6 The Africa Genomics Center and Consultancy, Nairobi, Kenya; 7 University of South Africa, Pretoria, South Africa; 8 Illumina - AMEA - Emerging Markets, Lagos, Nigeria; 9 Biotechnology School, Nile University, Giza, Egypt and Genetics Department, Faculty of Agriculture, Ain Shams University, Cairo, Egypt; 10 Higher Institute of Applied Biological Sciences of Tunis, University of Tunis El Manar, Tunis, Tunisia; 11 Inqaba Biotec East Africa Ltd, Nairobi, Kenya; 12 Institut Pasteur du Maroc, Casablanca, Morocco; 13 Department of Applied Sciences, Faculty of Health and Life Sciences, Northumbria University, Newcastle-upon-Tyne, England, UK; 14 University of KwaZulu Natal, Stellenbosch, South Africa; 15 Illumina - AMEA - Emerging Markets, Evry, France; 16 ISN Medical, Lagos, Nigeria; 17 Bio and Emerging Technology Institute, Addis Ababa, Ethiopia; 18 Bioinformatics Laboratory, College of Computing, University Mohammed VI Polytechnic, Benguerir, Morocco; 19 MyAfroDNA, Port Harcourt, Nigeria; 20 Biotechnology School, Nile University, Giza, Egypt; 21 Applied Genetics in Agriculture, Ecology and Public Health Laboratory, University of Tlemcen, Tlemcen, Algeria; 22 University of Port Harcourt, Port Harcourt, Nigeria; 23 Lakes and Fish Resources Protection and Development Agency (LFRPDA), Cairo, Egypt; 24 The Roslin Institute and the Centre for Tropical Livestock Genetics and Health, the University of Edinburgh, Edinburgh, UK; 25 Eppendorf Middle East and Africa FZ LLC, Dubai, United Arab Emirates; 26 Separations, Johannesburg, South Africa; 27 Laboratory of Biomedical Sciences, Food and Environmental Health (LaSBASE), Research Unit in Biomedical Sciences and Bioactive Substances (UR-2SB), Department of Biomedical Analysis (AMB), Higher School of Biological and Food Techniques (ESTBA), University of Lomé, Lomé, Togo; 28 Inqaba Biotec West Africa, Ibadan, Nigeria; 29 South African National Biodiversity Institute (SANBI), Pretoria, South Africa; 30 School of Medicine, University of Kinshasa, Kinshasa, Democratic Republic of Congo; 31 Kwame Nkrumah University of Science and Technology, Kumasi, Ghana; 32 North-West University, North West, Potchefstroom, South Africa; 33 Chemical and Biochemical Sciences-Green Process Engineering, Mohammed VI Polytechnic University, Benguerir, Morocco; 34 Department of Plant Sciences and the Bioeconomy, Rothamsted Research, Harpenden, UK; 35 Addis Ababa University, Addis Ababa, Ethiopia; 36 National Animal Genetic Resources Centre and Data Bank, Entebbe, Uganda; 37 University of Pretoria, Pretoria, South Africa; 38 Department of Biological Sciences, Elizade University, Ilara-Mokin, and A. P. Leventis Ornithological Research Institute, University of Jos, Jos, Nigeria; 39 Institute of Genomics and Global Health, Redeemer’s University, Ede, Nigeria; 40 Africa Biosystems Limited, Nairobi, Kenya; 41 Carl R. Woese Institute for Genomic Biology, Urbana, IL USA; 42 Agricultural Research Council, Biotechnology Platform, Pretoria, South Africa; 43 MGI-Tech, Shenzhen, China; 44 SequAna Core Facility, Department of Biology, University of Konstanz, Konstanz, Germany; 45 Institute of Genetics and Animal Biotechnology, Polish Academy of Sciences, Jastrzębiec, Poland; 46 Megaflex, Casablanca, Morocco; 47 Department of Plant Biology, Faculty of Science, University of Yaounde 1, Yaounde, Cameroon; 48 University of Calabar, Calabar, Nigeria; 49 Pwani University, Kilifi, Kenya; 50 Research & Conservation Support Society (RECOURSE), Kilifi, Kenya; 51 University of Glasgow, Glasgow, Scotland UK; 52 Biodec, National Biotechnology Research and Development Agency, Odi, Nigeria; 53 Department of Biology, Chemistry and Pharmacy, Freie University Berlin, Berlin, Germany; 54 International Institute of Tropical Agriculture, Ibadan, Nigeria and Institute for Biomedical Technologies, CNR, Bari, Italy; 55 University of Mauritius, Reduit, Mauritius; 56 Higher Institute of Biotechnology of Sidi Thabet, Tunis, Tunisia; 57 Department of Human Anatomy, University of Port Harcourt, Choba, Nigeria; 58 Claretian University of Nigeria, Nekede, Nigeria; 59 Department of Biomedical Sciences, Stellenbosch University, Stellenbosch, South Africa; 60 African Microbiome Institute, Division of Molecular Biology and Human Genetics, Department of Biomedical Sciences, Faculty of Medicine and Health Sciences, Stellenbosch University, Cape Town, South Africa; 61 Jomo Kenyatta University of Agriculture and Technology, Juja, Kenya; 62 National Defence University-Kenya, Nakuru, Kenya; 63 African BioGenome Project (AfricaBP), Juja, Kenya; 64 EMBL-European Bioinformatics Institute (EMBL-EBI), Hinxton, UK; 65 African Sustainable Agriculture Research Institute, Mohammed VI Polytechnic University, Laâyoune, Morocco

**Keywords:** Computational biology and bioinformatics, Genetics, Molecular biology, Plant sciences, Zoology, Agriculture, Developing world, Scientific community, Ethics, Ecology, Biodiversity

## Abstract

The African BioGenome Project (AfricaBP) is a Pan-African initiative aimed at improving food systems and biodiversity conservation through genomics while ensuring equitable data sharing and benefits. The Open Institute is the knowledge exchange platform of the AfricaBP, which aims to bridge local knowledge gaps in biodiversity genomics and bioinformatics and enable infrastructural developments. In 2024, the AfricaBP Open Institute advanced this mission by organizing 31 workshops that attracted more than 3500 registered attendees across 50 African countries, provided training to 401 African researchers in genomics, bioinformatics, molecular biology, sample collections and biobanking, and ethical considerations, across all five African geographical regions involving 40 African and non-African organizations. These workshops provide insights on applications of biodiversity genomics and bioinformatics to the African bioeconomy, as well as hands-on training in sample collection and processing, genomics, bioinformatics, molecular biology, and gene editing. Here, we provide the current understanding of the applications of biodiversity genomics and bioinformatics to the African bioeconomy through synthetic reviews and presentations, including descriptions of 31 workshops organized as well as three fellowship programs delivered or launched by the AfricaBP Open Institute in collaboration with African and international institutions and industry partners. We review the current national bioeconomy strategies across Africa and the economic impact of sequencing African genomes locally, illustrated by a case study on the proposed 1000 Moroccan Genome Project. Key recommendations include integrating biodiversity genomics and bioinformatics into national bioeconomy strategies, leveraging genomics for sustainable bioeconomy growth, and expanding capacity-building initiatives across Africa.

## Background

The African BioGenome Project (AfricaBP) (https://africanbiogenome.org/) is a transformative, continent-wide initiative aimed at sequencing, cataloging, and studying the genomes of Africa’s rich and diverse biodiversity. Among its ambitious goals is the sequencing of approximately 105,000 non-human genomes (plants, animals, fungi, protozoa, and other eukaryotes), a crucial endeavor to benefit the African population in areas of food security and biodiversity conservation with the applications of advanced tools of biotechnology and genomics to build a sustainable bioeconomy^1^. The Open Institute for Genomics and Bioinformatics (Open Institute) is the knowledge exchange platform of the AfricaBP, which was established to enable curriculum development, technology and infrastructure advancements, policy influence, encourage scientific entrepreneurship and enhance bioeconomy through biodiversity genomics and bioinformatics across Africa^2,3^.

The increasing concern about changing climatic conditions and their effects on the management and conservation of biological resources highlights the urgent need to develop effective models for sustainable resource management^4^. The bioeconomy is the production, utilization, and conservation of biological resources, including related knowledge, science, technology, and innovation, to provide information, products, processes, and services across all economic sectors, aiming toward a sustainable economy^5^. It entails, amongst others, the production of renewable biological resources and the conversion of these resources into value-added products^4,6^. According to the World Bioeconomy Forum, the current total value of the global bioeconomy is estimated at US$4 trillion^7,8^, encompassing the financial value of products in, amongst others, agriculture, forestry, food, bioenergy, biotechnology and green chemistry, which were exported worldwide^9^. This value could potentially increase to US$30 trillion by 2050, representing one-third of the global economic value^7,8^. Currently, the United States of America and North-West Europe lead in bioeconomy research^10^, with other regions, including Africa, lagging behind.

**Fig. 1:**
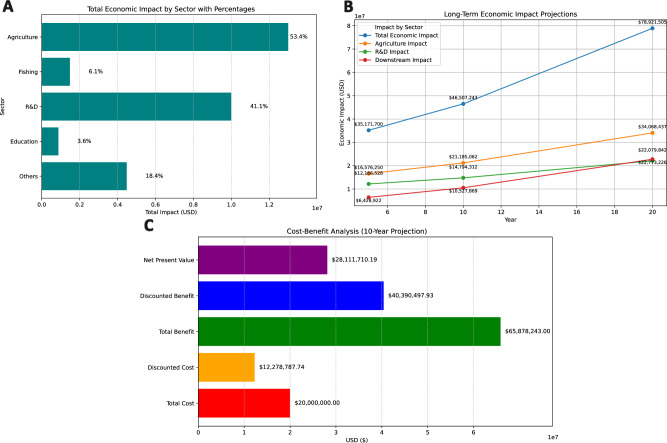
The proposed 1000 Moroccan genome project will create a positive economic impact and demonstrate an increased cost–benefit ratio. The predicted economic impact and cost–benefit analysis of the genomes project illustrate the projected economic benefits, long-term impacts, and cost–benefit analysis outcomes of the proposed 1000 Moroccan genomes project. **A** *Economic Impact by Sector:* shows the distribution of economic impacts across key sectors, including agriculture, fishing, research and development (R&D), education, and other sectors. The largest impacts are observed in agriculture and R&D, respectively, reflecting their central role in generating economic benefits from genome sequencing. The average agricultural contribution is 53% (the total economic impact attributed to agriculture was US$13 million, while the combined impact of all sectors amounted to ~US$24,340,000 million). The average R&D contribution is 40% (the total economic impact for R&D was $10 million, while the same overall economic impact of US$24,340,000 million). **B** *Long-term Economic Impact Projections:* illustrate the cumulative economic benefits over a 5-, 10-, and 20-year period. Sectors such as agriculture, R&D, and downstream industries exhibit significant growth, with total impacts surpassing $78 million after 20 years. This underscores the long-term sustainability of the genome sequencing investment. **C** *Cost–benefit Analysis (10-year Projection)*: displays the results of the 10-year cost-benefit analysis. It highlights key metrics, including Total Cost, Discounted Cost, Total Benefit, Discounted Benefit, and net present value (NPV). The analysis reveals a Benefit–Cost ratio (BCR) of 3.29, indicating that every dollar invested generates US$3.29 in benefits, affirming the economic viability of the project.

The bioeconomy is fundamentally dependent on biodiversity^7^, which provides megadiverse regions such as Africa the opportunities to increase national revenues in a post-fossil economy through genomics advances in generating microbial factories that produce important compounds for the chemical industry^11^ such as biorefineries^12^ (Fig. 1). However, of the 17 sectors included in bioeconomy strategies and monitoring across seven (7) countries and the European Union, genomics was notably absent^13^.

In Africa, efforts to implement biotechnology, genomics, bioinformatics, and computational biology such as the Southern African Biosciences Network and Biosciences East and Central Africa are contributing towards the growth of the African bioeconomy ecosystem, but these are variable across countries and regions^2,3,14–16^. For example, an analysis of 152 studies on the African bioeconomy showed South Africa as the leading nation in African bioeconomy research, with strong interconnection to other African geographical regions, followed by the gradual progress of Nigeria, Kenya, Ghana, as well as the slow progress of Tanzania, Ethiopia, Zimbabwe, Botswana, Rwanda, Egypt, and Madagascar^17^. The findings of the analysis contextualize continental research loopholes such as limited opportunities in prioritizing research and development (R&D) in public policies and strategies, including limited agricultural interventions to address natural resource degradation, climate change^18^ and limited information on the revenue value of the African bioeconomy^19,20^ as well as the contributions of biodiversity genomics and bioinformatics to the African bioeconomy^1^.

The AfricaBP Open Institute is enhancing capacity-building and strengthening efforts across Africa, advancing our knowledge in genomics and bioinformatics and their contributions to the African bioeconomy. For instance, in 2023, the AfricaBP Open Institute organized the inaugural edition of its regional workshop model involving awareness and hands-on practicals in genomics, bioinformatics, sample collections, and policy thematic areas^2,3^. In 2024, the AfricaBP Open Institute built upon its inaugural regional workshop model by organizing thirty-one (31) workshops through public–private partnerships, which attracted 3595 registered attendees across 50 African countries and trained 401 African researchers (Figs. S1–S4).

Here, we highlight the current understanding of the applications of biodiversity genomics and bioinformatics in driving the African bioeconomy; assess and predict the economic impact of locally sequencing genomes of African endemic and indigenous species and integrating sequencing into national economic plans and bioeconomy strategies; examine the advancements in the AfricaBP Open Institute in 2024 through the development of fellowship programs and its hands-on genomics and bioinformatics practical workshops; and provide actionable strategies for future directions of the AfricaBP Open Institute to drive measurable impacts.

## The predicted economic impact of biodiversity genomics on the African bioeconomy: A Moroccan case study

The global genomics market is projected to grow from US$42.64 billion in 2024 to US$66.85 billion by 2029, and it is driven by factors such as increased prevalence of human genetic disorders, rising adoption of personalized medicine, and technological advancements in genome sequencing^21^. In Africa, the economic impact of genomics and bioinformatics is significant. A cost–benefit analysis of the South African Beef Genomics Program revealed that a total investment of US$44 million over 10 years was expected to yield at least US$139 million in benefits, with an economic return of 18.70% and a Benefit–Cost Ratio of 3.1, indicating that the present value of future benefits would be approximately three times the costs incurred^22^. However, the genomics economy in Africa faces unique challenges, and it is yet to be maximized^23,24^. African countries allocate an average of 0.45% of their GDP to R&D^25^, significantly below the global average of 1.7%^26^. This underinvestment is largely attributed to insufficient government funding for R&D, infrastructure development, and human capital capacity-building initiatives^27^. These limitations hinder the transformation of Africa’s intellectual capital into tangible products and services that could boost economic growth^28^.

Biodiversity genomics offers transformative economic opportunities for Africa, with the sequencing of 105,000 species projected to generate substantial direct, indirect, and induced impacts across the continent. Contributing to these sequenced genomes requires concerted efforts at the national level, especially driven by national networks of scientists in biodiversity genomics and bioinformatics^1^. Here, we draw insights from Morocco’s proposed 1000 genome sequencing investment model (Fig. 1), highlighting the economic and societal benefits of genome sequencing for the African bioeconomy.

### Approaches and outcomes

We performed an economic impact and cost-benefit analysis (see Box 1 for detailed methodology) of sequencing 1000 Moroccan genomes locally in Morocco. To evaluate the economic impacts, an Input/Output (I/O) analysis was conducted using the Leontief I/O model^29^. This methodology examines economic interdependencies between sectors and quantifies the direct, indirect, and induced effects of investments. The Cost-Benefit Analysis framework assessed the economic feasibility and sustainability of the genome sequencing initiative over a 10-year period, and it involved using the economic metrics derived from I/O model outputs, environmental data, net present value calculations, and key assumptions such as an investment of US$20 million in the proposed 1000 Moroccan genome project. The outcomes show impacts across sectors, long-term projects, and economic returns as described below

- **Sectoral contributions agriculture and beyond**
  Genome sequencing directly benefits agriculture, fishing, research and development (R&D), and education (Fig. 1A). Agriculture, the largest contributor, accounts for over 53% of total economic output, driven by innovations like precision breeding of drought-resistant crops and disease-resilient livestock. These advancements improve yields and food security, especially for biodiversity-rich nations suchas Kenya and Madagascar^30,31^. Fishing and aquaculture also benefit through sustainable practices and resilient species identification (Fig. 1A). Meanwhile, R&D catalyzes innovation across agriculture, pharmaceuticals, and conservation, contributing 40% of economic returns (Fig. 1A). Educational investments ensure a skilled workforce capable of implementing these genomic advancements.
- **Long-term projections**
  The economic benefits of genome sequencing are projected to grow significantly over time (Fig. 1B). Over two decades, total economiccontributions are expected to rise from US$35 million in the first 5 years to US$79 million by the 20th year. Agriculture remains thedominant sector, reaching US$34 million by the 20th year, while R&D maintains a steady 28% contribution. Spillover benefits fromagriculture bolster downstream industries like food processing and ecotourism, collectively adding millions to Africa’s economy (Fig. 1B).
- **Cost-effectiveness: Strong economic returns**
  A cost-benefit analysis highlights the initiative’s economic feasibility, with a Benefit–Cost Ratio (BCR) of 3.29, indicating US3.29 in benefits for every US$1 invested (Fig. 1C). Over 10 years, a $20 million investment is projected to yield discounted benefits of US$40 million and a net present value (NPV) of US$28 million (Fig. 1C).

Box 1 **Materials and methods to estimate the economic impacts and cost–benefit of sequencing biodiversity in Africa: A Moroccan case study**

*Data sources*: The Supply and Use Table (SUT) was obtained from https://www.hcp.ma/, which is the Higher Planning Commission in Morocco, an independent government statistical institution. The input–output matrix was derived and kindly provided by Elguellab and Ezzahid, 2023^163^. The input–output matrix provides a comprehensive framework that outlines: (a) Inputs that involve goods and services consumed by industries for production, (b) Outputs that involve goods and services produced by each industry and their final destinations, such as exports, household consumption, or investments, and (c) Inter-industry flows which involve transactions showing how outputs of one industry serve as inputs for another. Analysis was performed by feeding these data into a custom Python script.

*Input/Output (I/O) analysis*: To evaluate the economic impacts of sequencing 1000 genomes in Morocco, an Input/Output (I/O) analysis was conducted using the Leontief I/O model. This methodology examines economic interdependencies between sectors and quantifies the direct, indirect, and induced effects of investments.

*Matrix structure*: A 27-sector I/O matrix was developed, encompassing agriculture, fishing, manufacturing, R&D, and services. Base case: Represented Morocco’s current economic structure without genome sequencing. Adjusted case: Integrated genome sequencing impacts, with increased technical coefficients for agriculture, fishing, and R&D sectors.

*Technical coefficients calculation:*

*A*_*ij* = Intermediate consumption by sector *i* from sector *j*/total output of sector *j*

*Model implementation:*

*Leontief inverse calculation:* (*I*−*A*)^−1^, where *A* is the technical coefficient matrix. Final demand vector (*F*): Allocated a $20M investment equally between R&D and agriculture to simulate genome sequencing impacts.

*Adjustments for productivity:* A 10% productivity improvement was applied to agriculture-related sectors. Strengthened linkages were modeled between R&D and other sectors, including education, fishing, and agriculture.

**Cost–benefit analysis (CBA)**

The CBA framework assessed the economic feasibility and sustainability of the genome sequencing initiative over a 10-year period.

*Data sources:* Economic metrics derived from I/O model outputs. Environmental data included reductions in carbon emissions, land use, and water consumption. Export data leveraged trends in the agricultural, aquaculture, and pharmaceutical sectors.

*Analytical framework:* Net present value (NPV): Calculated as

NPV = Σ(Benefits_*t*−Costs_*t*)/(1 + *r*)^t^

where *r* = 5% (discount rate). Benefit–Cost ratio (BCR): Determined by dividing total discounted benefits by discounted costs.

*Key assumptions:* Investment costs: $20M, including operational and infrastructural expenses. Productivity gains: Derived from sectoral multipliers established in the I/O analysis.

Box 2 **Participants’ feedback**

The participants’ feedback was overwhelmingly positive (Fig. S5). Approximately 90% of attendees agreed that the workshops met their expectations, with strong support for organizing similar initiatives in the future. Additionally, 75% of participants reported a significant improvement in their knowledge of biodiversity genomics, and over 40% found the content engaging, helpful, and appropriately structured. This shows the workshops’ success in delivering valuable content and addressing the educational needs of participants from diverse scientific backgrounds. Feedback on practical sessions showed that nearly 55% of participants found them straightforward and easy to follow, with 50% expressing overall satisfaction and 37% rating their experience as good.

While these results are encouraging, participants pointed out areas for improvement. Technical issues, particularly network reliability, were highlighted as significant barriers. The word cloud analysis highlighted these concerns, with “time” and “network” being common themes. The workshops aimed to provide foundational exposures, and according to survey respondents, the limited time allocated to practicals constrained the depth and comprehensiveness of the sessions. This could explain participants noting certain gaps between their expectations and the workshop content. Some attendees hoped for a more integrated approach that combines theory with hands-on bioinformatics training after sequencing.

The practical session at the University of Yaoundé 1 was canceled at the start of the regional workshop, highlighting the risks of over-reliance on a single sponsor and stressing the importance of diversifying funding sources, including local, institutional, and national contributions. Solutions like transitioning to electronic and virtual formats, as demonstrated by the University of South Africa’s successful adaptation of its Ethics, Law, and Social Implications (ELSI) of Genetic Research practicals, can help maintain program continuity when in-person congregation fails. Despite these challenges, sentiment analysis reflected a positive outlook overall, with participants describing the workshops as an “excellent effort” and showing strong motivation for future engagement.

### Integrating genomics and bioinformatics into national economic plans and bioeconomy strategies

To fully harness the transformative potential of biodiversity genomics, African countries and institutions should adopt a strategic and integrative economic approach. Establishing regional sequencing hubs and state-of-the-art laboratories is critical to reducing dependency on external facilities, fostering local innovations, and providing jobs for early careers and established scientists^32,33^. Simultaneously, capacity-building initiatives should be strengthened through comprehensive training programs and international partnerships to equip a skilled workforce with the expertise needed to implement genomic technologies effectively^2,3^. Prioritizing the sequencing of high-impact species, such as drought-resistant crops and disease-resilient livestock, will ensure maximum economic, environmental, and societal benefits^34^. Furthermore, robust policies must be developed to safeguard genomic data, promote equitable sharing of benefits, and protect intellectual property derived from genomic research^35^. Encouraging public–private partnerships through incentives like tax breaks and grants will attract investments and enable collaboration between governments, academia, and industries^36^.

## The landscape of national bioeconomy strategies across Africa

African nations are increasingly adopting bioeconomy strategies to govern biodiversity for sustainable development, addressing gaps in education, R&D, and policymaking^17^. Only 12 African countries have established bioeconomy strategies: South Africa and Ethiopia have comprehensive bioeconomy strategies^37,38^, while Senegal, Nigeria, Ghana, Mali, Kenya, Mauritius, Mozambique, Namibia, Tanzania, and Uganda have bioeconomy-related strategies^37^.

South Africa is a continental leader in the bioeconomy, integrating agriculture, health, and industry within its bioeconomy framework through institutions like the Technology Innovation Agency and significant public-private investments^39,40^. Notably, sustainable marine aquaculture has become an emerging component of this food security and economic growth strategy, aligning with broader national goals to diversify the blue economy and improve nutrition, employment, and livelihoods through aquaculture innovation^41,42^. One example is the application of genomic techniques such as quantitative trait loci (QTL) mapping and genome annotation to improve growth, disease resistance, and productivity in key aquaculture species like abalone (*Haliotis midae*)^43,44^. Genomic studies on Cape hakes also reveal population structure shaped by overfishing, prompting region-specific management^45,46^. These efforts are supported by regional initiatives such as ASTRAL and LIMAQUA, which promote the integration of genomics into sustainable aquaculture practices across Africa^47,48^. Collectively, these initiatives have positioned the country as a global bioeconomy model, supported by strong policies and infrastructure^39^.

Similarly, within the Southern African region, Namibia links its bioeconomy development strategy to its Vision 2030 by focusing on biomass from bush thinning for job creation, biodiversity management, and sustainable land use, all established by multi-stakeholder engagement^17,39,40^. In East Africa, the 2022 modern bioeconomy strategy by the East African Community, which includes Burundi, Kenya, Rwanda, South Sudan, Tanzania, and Uganda, has significant potential to support several critical development goals and targets for the region. This strategy aimed at creating an enabling environment for increased Science, Technology, and Innovation (STI) investments to support sustainable development and socio-economic transformation^49^. For instance, Uganda has particularly focused its bioeconomy development strategy on advancing its agricultural and energy sectors through biotechnology and nanotechnology, and also on promoting public-private partnerships^39,40^. Furthermore, Ethiopia has recently implemented a national bioeconomy strategy to benefit from its rich diversity of flora and fauna through the development and promotion of a sustainable knowledge-based bioeconomy^38^.

In West Africa, Nigeria aligns its bioeconomy initiatives with its national biotechnology policy (2001) and the biofuel policy incentives (2007)^20^, while Ghana aligns with the Climate-Smart Agriculture Plan^39^, respectively. Ghana capitalizes on its rich biodiversity and biomass resources to promote economic growth, including the development of sustainable bio-manufacturing industries^40^. Moreover, educational institutions and public–private partnerships play a central role in driving Ghana’s science and technology outputs for economic growth^50^.

## Current understanding of African bioeconomy through biodiversity genomics

The AfricaBP Open Institute regional workshops in 2024 were organized using the model previously discussed in Sharaf et al.^3^, over a 5-day period. Days 1 and 2 were symposium style (Fig. S1) which recorded a total of 3595 registered attendees (Figs. S2–S4) while Day 3–5 were distributed practicals across multiple sites and countries. The regional workshops were coordinated by the University of Port Harcourt, Nigeria (West Africa) from 5 to 9 May, Jomo Kenyatta University of Agriculture and Technology (JKUAT), Kenya (East and Central Africa) from 5 to 9 August, University of South Africa (Southern Africa), from 9th to 13th September, and Mohammed V University in Rabat (UM5R), Morocco (North Africa) from 25 to 29 November, respectively. Here, we provide a synthetic review of selected keynotes, guests, oral and poster presentations by early career and established researchers during Day 1 and Day 2 across all five African geographical regions, and these are categorized under the five AfricaBP Grand Challenges^1,2^ as given below.

- Genomics and bioinformatics technologies for the agri-environment
  Recent advancements in genomics and bioinformatics have the potential to revolutionize agriculture, livestock management, and biodiversity conservation^51,52^. For instance, progress in sequencing technologies, particularly long-read sequencing methods, has addressed challenges in genome assembly, enabling near error-free reference genomes that significantly enhance the accuracy of functional genomic analyses^53^.
  Furthermore, the generation of high-quality genome assemblies for African cattle breeds, namely: *Bos indicus* *×* *Bos taurus Mpwapwa*, *Bos indicus Iringa Red*, *Bos indicus Singida White*, *Bos indicus Gudali*, and *Bos taurus Lagune*, represent a significant step forward towards understanding the genetic diversity and adaptive traits underlying Africa’s extensive cattle heritage^54–56^. These assemblies provide resources for identifying genetic markers linked to selection signatures and adaptive expression quantitative trait loci (eQTLs)^57^. Moreover, they facilitate the development of breeding programs designed to enhance climate resilience and disease resistance, ensuring sustainable livestock systems for the continent^58,59^.
  The applications extend beyond livestock genomics. The genome assembly of *Hippobosca camelina*, commonly known as the camel ked, revealed chemosensory genes that are vital to its parasitic lifecycle, enabling targeted control strategies to mitigate economic losses in camel-rearing communities^60^. Similarly, biodiversity research utilizes genomic tools, with DNA barcoding successfully resolving hidden species within the *Labeobarbus* genus, advancing both conservation efforts and sustainable fisheries management^61^.
- Crops and livestock improvements and health
  Africa is making significant advancements in genomics and innovation to enhance agricultural productivity and address challenges in crop and livestock health for food security. These advancements are evident in several initiatives such as the West African Virus Epidemiology (WAVE) program in Ivory Coast, a collaborative effort being conducted across 10 West and Central African countries^62^, illustrating the power of genomic surveillance in managing plant pathogens and controlling the spread of exotic diseases^63^. In Nigeria, genetic studies on rice have identified key traits linked to yield stability and environmental resilience, achieved through marker-assisted backcrossing^64^. This progress is mirrored in other African contexts, such as in Morocco, where the exploration of *Argania spinosa* has led to valuable understanding of functional genes involved in oil quality and stress resistance^65^. Similarly in Ethiopia, comprehensive genomic analyses of sorghum are revealing key genetic loci associated with the development of robust root systems, a critical adaptation for drought tolerance^66^. These advancements extend beyond crop species. In Tanzania, dairy cows are demonstrating enhanced resilience to high temperatures, which is due to the identification of genetic markers associated with thermal tolerance through genomic studies^67^. Furthermore, in Morocco, the genetic makeup of the local Sardi sheep breed is characterized, enabling the development of targeted breeding programs that meet the specific needs of local livestock producers^68,69^. Additionally, research from Morocco highlights the important role of both neutral and adaptive genomic diversity in providing resilience to environmental pressures, laying the groundwork for sustainable breeding programs^70,71^. The integration of genomics with Artificial intelligence technologies is being actively applied in breeding programs, aiming to enhance food security by prioritizing biodiversity, improving resilience, and optimizing agricultural practices^72^.
- Genomics for the conservation of endangered and endemic species
  Over 90% of endangered, endemic, and indigenous African species remain unsequenced, despite their ecological importance and declining populations^1^. Genomic research on endemic and endangered African species provides important understanding for their preservation and conservation across Africa^73^. For instance, genomic analyses of African wildlife have demonstrated how disruptions in migratory routes and introgression events shape population structures and phylogeography^74,75^. Another example is the employment of DNA barcoding techniques by the South African National Biodiversity Institute (SANBI) at the Pretoria National Botanical Gardens to protect South Africa’s biodiversity hotspot^76^. Advanced methods such as remote sensing and machine learning, are used to study plant phenology, including flowering patterns and their associations with insect pollinators^77^. Additionally, studies using Malaise traps in cultivated and natural areas are used to reveal variations in insect diversity, while Natural Language Processing techniques have been applied to classify insect functional groups, enhancing the understanding of plant–insect interactions and their ecological significance^78^.
  Furthermore, the Institute of Genomics and Global Health (IGH, formerly the African Centre of Excellence for Genomics of Infectious Diseases—ACEGID) at Redeemer’s University, Nigeria, employs genomic techniques to address microbial threats in West Africa. This includes the use of real-time genomic sequencing during outbreaks, as evidenced during the Ebola and COVID-19 epidemics^79,80^. Notable advancements include the development of a multiplex testing platform capable of detecting up to 49 pathogen species in a single test and genome-wide association studies (GWAS), identifying genes linked to resistance and susceptibility to diseases like Lassa fever^81^. These efforts enhance health crisis management and mitigate risks to both human and wildlife populations. Genomic tools also facilitate the identification of cryptic species, including those critically endangered^82^. Understanding adaptive traits provides opportunities for timely interventions during disease outbreaks, population declines, or the emergence of invasive species. For example, research on giraffe populations identified significant genetic differentiation and historical gene flow among lineages, challenging traditional species classifications and enriching our understanding of their evolutionary history^83^. These diverse applications of genomics enable the monitoring of species populations and their health, driving the development of targeted conservation strategies, therefore, preserving Africa’s unique genetic resources and ecological heritage^84–86^.
- Ethics, socio-economics, and policy issues
  Africa, the world’s second most biodiverse continent^87^, holds immense potential for aligning biodiversity conservation with socio-economic development, thereby advancing towards a green and blue economy^88,89^. Preserving ecological infrastructure is crucial for maintaining ecosystem services and reducing reliance on built infrastructure. For example, South Africa’s healthy catchments are vital for water resource management, indicating the significance of ecological health in ensuring resource resilience^90^. Similarly, Nigeria’s National Biosafety Management Agency (NBMA) reinforces its commitment to safeguarding biodiversity through the responsible application of modern biotechnology^91^.
  Global frameworks, such as the Kunming–Montreal Global Biodiversity Framework adopted by the Convention on Biological Diversity^92^, and supported by the International Union for Conservation of Nature (IUCN), offer structured pathways to halt and reverse biodiversity loss and integrate conservation into global policy, focusing on biodiversity preservation and the enhancement of ecosystem services for present and future generations^93^.
  Data-driven strategies are equally important; the Global Biodiversity Information Facility (GBIF), for instance, mobilizes extensive biodiversity records to provide open access to data, empowering policymakers and researchers to design sustainable, evidence-based practices^94^. African organizations and programs such as the Nigerian Conservation Foundation contribute to (and leverage on) the GBIF platform to promote sustainable practices in agriculture, forestry, and fisheries, while quantifying the bioeconomy across the region^95^ and supporting IUCN’s agenda. This approach strengthens Africa’s bioeconomy by advancing biodiversity informatics and guiding policies that promote sustainable resource use^96^.
  Efforts to promote sustainable landscape connectivity, such as those in the Maghreb region of North Africa, driven by UNESCO, enhance ecosystem resilience while providing socio-economic benefits like improved livelihoods and climate adaptation. Ethical governance and inclusive policies are central to these initiatives^97,98^.
  Collectively, these initiatives, integrating cultural and biological diversity into science and education, align with the AfricaBP Open Institute’s local-first, global-later strategy. This strategy is founded on regional training, improved data sharing, and inclusive policy advocacy^1–3^ and prioritizes the incorporation of local ethics and socio-economic policies within international discourse^2^, thereby advancing Africa towards a sustainable path for biodiversity and socio-economic management.
- Technology development, knowledge exchange, industry, and commercialization
  Many sectors, such as agriculture, construction, food, and beverage, rely on resources from the ecosystem, with a decline in the ecosystem functionality estimated to cost the global economy more than US$5 trillion a year^99^. Opportunities exist to innovate around conservation, restoration, and building for resilience, integrating genomics, computing, and engineering. The Woolly Mammoth de-extinction project supported by Colossal aims at restoring Alaska’s forests by assessing the extent to which contemporary Arctic ecosystems are conducive to the rewilding of megaherbivores, using a woolly mammoth (*Mammuthus primigenius*) proxy as a model species through CRISPR-Cas9^100^, is one such example, and its economic impact is likely to be felt in the recreational sector^101^. In Africa, similar efforts could aim to restore ecological balance and biodiversity, which, in turn, drive sustainable industries and stimulate economic growth. For example, biodiversity conservation contributes to food security through pollinators, helps stamp out disease reservoirs, and supports medicinal innovations, such as anticancer drugs^102,103^. The protection of endemic species through strong intellectual property frameworks is also evolving across Africa. Such frameworks allow for the commercialization of genetic resources, protect local innovations, and encourage investments in the bioeconomy, especially since intellectual property protection is mandatory in facilitating open science practices, promoting collaboration, and enabling innovation, which encourages startups^104^. Similarly, progress in entrepreneurship has mainly been driven by the private sector in Africa^105^. However, there is a need to take an integrative, cross-disciplinary and multi-sectoral approach to advance opportunities in the bioeconomy, including academia, industry, government, policymakers, where scientific knowledge can be leveraged not only for economic gains but for broader societal impact such as building pathways that empower the translation of research to market-ready solutions.

## Summary of course content of selected practical workshops

The diversity of hands-on workshops (Fig. S1 and Table 1) conducted as part of the AfricaBP Open Institute regional workshops 2024 highlights the continent’s advancing capabilities in biodiversity genomics and bioinformatics (Box 2). Days 3–5 were focused on hands-on practicals to provide foundational exposures in genomics, bioinformatics, sample collections, ethics, and genome editing applicable to non-human species and hosted simultaneously across multiple sites during each scheduled regional workshop across all five African geographical regions. The AfricaBP brokered opportunities with 40 African institutions and industry partners to organize twenty-seven (27) hands-on practical workshops to introduce participants to cutting-edge technologies in genome sequencing, bioinformatics, sample collections, ethics, and genome editing, which trained 401 African researchers (see Table 1 for details, Fig. S1).

**Table 1:** Summary of course contents of selected practical workshops during the practical sessions of the AfricaBP Open Institute regional workshops 2024

S/N	Practical session thematic area	AfricaBP regional workshop	Name of practical host organization and country of affiliation	Title of hands-on practical session held	Summary of course content
1	Genome editing	North Africa	African Genome Center, University Mohammed VI Polytechnic, Morocco	CRISPR/Cas9 fundamentals: A comprehensive guide to designing genome editing experiments in plant and animal biology	The workshop was hosted by the African Genome Center and provided a comprehensive introduction to CRISPR/Cas9 technology and its applications in plant and animal biology. Theoretical sessions covered the basics of CRISPR/Cas9 (Clustered Regularly Interspaced Short Palindromic Repeats/CRISPR-associated protein 9), including its mechanism, applications in research, and how to do a blast search for a particular gene and to design efficient guide RNAs (gRNAs). In the practical sessions, participants received hands-on training in cloning-based CRISPR technique using tomato (*Solanum lycopersicum*) and rice (*Oryza sativa*) gRNAs targeting *SlLOB1* and *OsS5H*, respectively, and cloning-free CRISPR technique on mouse and bovine genomic DNA, allowing them to practice different approaches and understand how to adapt CRISPR in their research. Participants were also trained to evaluate editing efficiency and accuracy by analyzing CRISPR-edited samples. Additionally, the latest CRISPR technologies and their applications in research and industry were highlighted by industry experts from Integrated DNA Technologies, ThermoFisher Scientific, and Eppendorf.
2	Genome sequencing and bioinformatics analysis	North Africa	Institut Pasteur du Maroc, Morocco	Unlocking the power of genomics: Advancing whole genome sequencing with Illumina in Morocco	The workshop was hosted by Institut Pasteur du Maroc in collaboration with Illumina, and its Morocco-based partner, Megaflex. Participants engaged in the full workflow of high-throughput sequencing using the Illumina NextSeq 2000 platform. Practical sessions covered sample preparation and DNA extraction, library preparation, whole genome sequencing of two indigenous plants, the Argan tree (*Argania spinosa*) and Caroub tree (*Ceratonia siliqua)*, and bioinformatics analysis of the generated data. The workshop provided a platform for participants to enhance their skills, discuss research challenges, and explore collaborative opportunities.
3	Genome sequencing and bioinformatics analysis	Southern Africa	North-West University (NWU), South Africa	NWU Oxford Nanopore practical workshop supported by DIPLOMICS and Whitehead Scientific	The practical workshop hosted by North-West University and supported by Whitehead Scientific, Oxford Nanopore Technologies (ONT) and DIPLOMICS, trained participants in sequencing technologies and applications, focusing on ONT. The workshop began with lectures on sequencing applications, project planning, and cost considerations, followed by sessions introducing online resources and the 1KSA project^122^. Participants performed library preparation on *Dicerothamnus rhinocerotis* and *Klebsiella pneumoniae* using the Native Barcoding Kit, applying quality control with NanoDrop and Qubit fluorometer and calculating sample and library dilutions before loading flow cells for sequencing on the MinION platform. Data analysis was conducted using a GUI and Linux-based pipelines. On Day 3, participants learned about washing and re-using flow cells, data storage, and publishing/sharing research outputs.
4	Genome sequencing and bioinformatics analysis	North Africa	Higher Institute of Applied Biological Sciences of Tunis- University of Tunis El Manar, Tunisia	Molecular biology techniques and bioinformatics applied to microbiology—characterization of microbial diversity using metabarcoding	The practical workshop, hosted at ISSBAT, University of Tunis El Manar, provided hands-on training in molecular biology and bioinformatics applied to microbiology. Day 1 covered bacterial DNA extraction, using the boiling method for pure culture of Gram-negative bacteria^123^ and a commercial DNA extraction kit for the total ruminal bacterial community. DNA quality assessment via Nanodrop and gel electrophoresis, amplification of 16S rRNA genes using PCR, verification of PCR products by agarose gel electrophoresis were performed, and phylogenetic tree construction was done in MEGA using sequence alignment and evolutionary models^124^. Day 2 focused on preparing genomic DNA libraries for MinION sequencing platform, involving DNA end-repair, A-tailing, adapter ligation, quality assessment using Qubit^125^, and loading libraries onto the MinION Flow Cell. Day 3 introduced metabarcoding for microbial diversity studies, including demultiplexing sequencing reads, generating OTUs/ASVs with clustering algorithms, alpha and beta diversity analyses, and using bioinformatics tools in R statistical software for metagenomic data analysis^110^.
5	Genome sequencing and bioinformatics analysis	Southern Africa	Separations, South Africa	AfricaBP Open Institute Illumina sequencing technologies practical session—Hosted by Separations	Hosted by Illumina and its South Africa-based partner, Separations, this workshop trained participants on whole-genome sequencing library preparation and genome assembly using Illumina sequencing-by-synthesis technology. Genomic DNA was extracted from Nguni cattle, an indigenous South African breed, which is considered a sub-type of Sanga (*Bos taurus Africanus*), using the Macherey Nagel NucleoMag® Blood 200 μL kit. Library preparation was performed with 500 ng DNA using the Illumina DNA PCR-Free Prep Tagmentation workflow. The resulting libraries were quantified, pooled by mass, and sequenced on the Illumina NovaSeq 6000 system with a 2 × 150 bp configuration. The genome assembly used a reference-guided approach based on the *Bos taurus* and *Bos arnee* genomes. Reads were trimmed for quality and mapped to the bosTau9 reference genome using the Illumina DRAGEN Bio-IT Platform. De novo assembly was performed with Megahit^126^, yielding a draft Nguni genome with 2.38 Gb, 391,467 contigs, and an N50 range from 25,291 to 7832 bp.
6	Genome sequencing and bioinformatics analysis	East and Central Africa	Inqaba Biotec East Africa (IBEA)	Sanger sequencing technology in species barcoding	The workshop provided a comprehensive introduction to DNA sequencing and analysis techniques. Lectures covered next-generation sequencing, Sanger sequencing, and molecular identification of diverse organisms, including bacteria, sessile metazoans, and plant species. Practical sessions included DNA extraction from sponges (*Clathria reinwardti*, *Lissodendoryx hawaiiana, and Callyspongia subarmigera*) and blood samples utilizing Zymo Research DNA extraction kits, followed by PCR amplification, gel electrophoresis, and Sanger sequencing data analysis. DNA concentration and purity were assessed using a NanoDrop fluorometer. Participants gained hands-on experience in laboratory techniques and bioinformatics tools. The workshop focused on the critical importance of sample preparation and quality control for the success of subsequent sequencing experiments.
7	Genome sequencing and bioinformatics analysis	Southern Africa	University of Pretoria, South Africa	AfricaBP Open Institute—DIPLOMICS—UPGL: Oxford Nanopore Technologies laboratory workshop	This workshop, in partnership with DIPLOMICS and Oxford Nanopore Technologies (ONT), focused on biodiversity through the DIPLOMICS South Africa 1KSA genome project^122^. The gDNA of five 1KSA species were used for training and include *Loxodonta africana, Arctocephalus p. pusillus, Lepus saxatilis, Lepus capensis*, and *Lupulella mesomelas*. In addition, *Cherax quadricarinatus* gDNA and the DNA from its gut microbiome, a commercially available microbial community and the ONT Lambda control sample were included as workshop training samples. Participants prepared barcoded sequencing libraries, which were sequenced on PromethION flow cells. Daily flow cell washes and reloading were performed. Principles of ONT technology, project planning considerations, and DNA quality control were introduced alongside a demonstration of ONT’s analysis software. DIPLOMICS also highlighted the 1KSA initiative aimed at sequencing 1000 South Africa’s biodiversity genomes. Small group discussions allowed participants to explore library preparation adapted to their research. The final session included a comprehensive review of sequencing results, an introduction to ONT’s online user community, and a presentation of UPGL’s and DIPLOMICS’ optimized methods to enhance sequencing output and quality.
8	Genome sequencing and bioinformatics analysis	West Africa	Institute of Genomics and Global Health (IGH, formerly African Centre of Excellence for Genomics of Infectious Diseases - ACEGID), Nigeria	Whole genome sequencing of the rodent genome on Illumina NextSeq2000	The workshop, hosted at the Institute of Genomics and Global Health (IGH, formerly ACEGID), focused on sequencing genomes of rodents (*Crocidura* spp.) using the Illumina PCR-Free Library Preparation Kit. Day 1 covered DNA extraction, theory, and practicals of Illumina DNA PCR-Free Library Prep, including DNA quantification, tagmentation, and amplification workflows. Day 2 included advanced library preparation steps, quality control using Qubit assay kits, pooling, and loading libraries onto the NextSeq 2000 sequencing system. Day 3 introduced BaseSpace Sequence Hub, followed by retrieving sequencing data, bioinformatics analysis, and demonstrations of its applications.
9	Genome sequencing and bioinformatics analysis	South Africa	Agricultural Research Council, South Africa	MGI showcasing and training on innovative stLFR technology at the African BioGenome Project workshop	Held at the Agricultural Research Council’s Biotechnology Platform (ARC-BTP) in Pretoria, South Africa, this workshop introduced participants to MGI’s innovative MGIEasy single-tube long fragment read (stLFR) technology. The training aimed to address the limitations of short-read sequencing by demonstrating the stLFR technology, which enables high-quality structural variant detection, haplotype phasing, and de novo assembly. The workshop combined theoretical sessions with wet lab hands-on training, covering MGI’s next-generation sequencing workflow. Participants engaged in library preparation, DNB making and circularization, sequencing, and data reporting using the DNBSEQ-G400 platform, achieving a sequencing QC metric of Q30 > 91% for all four lanes.
10	Genome sequencing and bioinformatics analysis	South Africa	Stellenbosch University, South Africa	From sample to sequencing: metagenomic sequencing on the ONT long-read platform	The workshop was hosted at the Pathology Research Facility, Stellenbosch University, with a combination of theoretical and practical sessions offered across three days. Participants were introduced to sample handling and best practices in designing metagenomic studies, including the importance of contamination control, and performed DNA extraction of human stool samples. DNA quality assessment was performed on both human and buffalo stool DNA, using both spectrophotometric and fluorometric assays. This was followed by sequencing library preparation and metagenomic sequencing on the Oxford Nanopore MinION long-read platform. Finally, participants were introduced to command-line computing environments, basic bioinformatics analysis, and quality assessment and analysis of long-read metagenomic sequencing data using the EPI2ME software suite.
11	Bioinformatics analysis only	North Africa	College of Computing, Mohammed VI Polytechnic University, Morocco	Annotation of repetitive DNA sequences in plant genomes	This workshop was hosted by the Bioinformatics Laboratory at UM6P and focused on repetitive DNA and its biological roles, combining theoretical lectures with hands-on training. The workshop covered tools and techniques for repeat annotation, simple sequence repeats (SSRs) analysis, and transposable elements (TEs) identification. Day 1 began with an overview of repetitive DNA, types, and roles in plant genomes. Participants were introduced to tools like RepeatModeler^127^ and RepeatMasker^128^, followed by practical sessions on building and utilizing repeat libraries using *Oryza sativa* Japonica group (NCBI RefSeq assembly: GCF_034140825.1) as a case study. Day 2 focused on SSRs, including their applications in plant genetics and breeding. Lectures covered SSR marker development, and participants used tools like MegaSSR^129^ and in silico PCR to identify and analyze SSRs using 50 *Oryza sativa* genomes for applications such as genetic diversity studies. On the final day, participants explored TEs and genome assembly quality assessment using tools like EDTA^130^, MegaLTR^131^, and PlantLAI^132^. The event concluded with an introduction to UM6P’s bioinformatics platform and its role in genomics research^133^.
12	Bioinformatics analysis only	North Africa	Faculty of Sciences, Mohammed V University in Rabat, Morocco	Machine Learning for biologists	The workshop introduced artificial intelligence and machine learning approaches in computational biology. The training focused on using BioPython for DNA sequence analysis^134^. Exercises targeted efficient DNA data and sequence retrieval and manipulation techniques, showing their application to genomics and other biological datasets. Additionally, the course involved supervised learning for predictive modeling, including multivariate linear regression and preprocessing techniques such as feature scaling and *Z*-score and mean normalization^135,136^. It also covered unsupervised learning and logistic regression for binary classification, with focus on sigmoid activation, optimization tuning, and iterative convergence^135^.
13	Bioinformatics analysis only	North Africa	Nile University, Egypt	Exploring bioinformatics in marine biology	The practical focused on bioinformatics applications in marine biology to address biodiversity loss. Training included biodiversity genomics, sequencing technologies, and epigenetic plasticity in marine conservation. Practical sessions involved quality assessment of long-read sequencing data, taxonomic classification using BLAST tools, and operational taxonomic unit (OTU) analysis. Additionally, participants explored alpha and beta diversity, tree algorithms, and organelle-targeted proteins.
14	Bioinformatics analysis only	North Africa	University of Abou Bekr Belkaid Tlemcen, Algeria	Bioinformatics analysis of RNA sequencing and SNP genotype data	The workshop focused on three key bioinformatics concepts. The Ion Torrent session introduced sequencing workflows, highlighting high-throughput efficiency and Unix navigation for genomic data handling. The RNA-seq session addressed data quality assessment, genome indexing, alignment file processing, gene expression quantification, differential gene analysis, and visualization techniques. Finally, the SNP session explored Single Nucleotide Polymorphisms (SNPs), covering data processing, quality control, and genome-wide analyses, including principal component analysis (PCA) and population structure analysis using ADMIXTURE^137^. All analyses were demonstrated using cattle data, offering practical insights into the application of these methods.
15	Bioinformatics analysis only	West Africa	Kwame Nkrumah University of Science and Technology, Ghana	Eukaryotic genome assembly and annotation workshop	This workshop focused on sequencing approaches and technologies for eukaryotic genome assembly and annotation using Next Generation Sequencing (NGS) data. Hands-on sessions were based on the whole genome sequence of *Tetraena mongolica*, sequenced on the PacBio HiFi platform^138^. The workshop covered file formats, quality control techniques, and methods for processing long-read data. It also included de novo genome assembly, genome quality assessment, and genome annotation, focusing on evidence-guided gene prediction and quality evaluation.
16	Bioinformatics analysis only	East & Central Africa	University of Kinshasa, Democratic Republic of the Congo	Genomic data analysis using Galaxy	The Genomic Data Analysis using Galaxy Workshop at the University of Kinshasa provided hands-on training focusing on bioinformatics tools for genomic analysis. Participants worked with DNA reads from *Agaricia lamarcki* reads generated by the Illumina NovaSeq 6000, sourced from the European Nucleotide Archive^139^. The workshop covered Trimmomatic for assessing sequencing read quality^140^, SPAdes for genome assembly^141^, and QUAST for evaluating assembly quality^142^. Participants gained practical experience in processing raw sequencing data, improving data accuracy, and conducting robust genomic analyses. Additionally, they explored Galaxy as a platform for genome annotation^143^, learning about tools for gene prediction, functional annotation, and other genomic processes.
17	Bioinformatics analysis only	East & Central Africa	National Animal Genetic Resources Centre & Data Bank, Uganda	Bioinformatics: Inference of genetic diversity and population structure from Sanger sequences	This workshop covered the analysis of genetic diversity and population structure using Sanger sequencing of the cytochrome c oxidase subunit I gene. It included multiple sequence alignment, phylogenetic tree construction, and haplotype network building using MAFFT^144^, MEGA^124^, DNaSP^145^, and popART^146^ applications. The training involved the analysis of original data from^147^ and ^148^, to understand concepts of founder and bottleneck effects, and species invasion, respectively. It also addressed the use of population genetic proxies, including migration (*N*_m_) and (*F*_ST_)^149^ to infer differentiation between populations. The focus was on bioinformatics applications for conservation and the interpretation of sequence analysis results.
18	Bioinformatics analysis only	East & Central Africa	Bio and Emerging Technology Institute (BETin), Ethiopia	NGS data analysis: From genome mapping to visualization	This workshop, hosted by BETin in collaboration with Addis Ababa University, Ethiopia, focused on introducing the role of genomics and bioinformatics in genetic resource conservation. It covered Next Generation Sequencing (NGS) data analysis, genome mapping, and visualization. Sessions included NGS data quality control, read mapping on reference genomes such as *Arabidopsis thaliana*, and analysis using open-source bioinformatics tools such as Burrows-Wheeler Alignment tool (BWA)^150^, SAM tools^151^, BCF tools^152^ and Integrated Genomic Viewer (IGV) for visualization^153^. The workshop also included training on the basic Linux environment for NGS data analysis.
19	Bioinformatics analysis only	East & Central Africa	Pwani University, Kilifi, Kenya (co-hosted by University of Copenhagen)	Introduction to population genomics in wildlife conservation	This workshop focused on how to use genomic data for population and conservation genomics in African wildlife species. We focused on presenting the principles behind different estimates and analyses, as well as their application, critical evaluation, and best practices. On Day 1, we covered an introduction to whole-genome sequencing data, SNP and genotype calling. On Day 2, we covered basic topics in population genetic analyses, i.e. the concepts of genetic diversity and inbreeding, and how to estimate them using genome-wide heterozygosity and runs of homozygosity. On Day 3, we proceeded to more complex analyses, including inferring population structure and admixture using Principal Component Analysis (PCA), ADMIXTURE^137^ and F_ST_^149^, and inferring gene flow using D-statistics^154^. The participants got hands-on exposure and tutoring in a range of different analyses software beyond those mentioned above, including PLINK^155^, evalAdmix^156^, vcfTools^113^ and R statistical software^110^. We also presented several case studies exemplifying how the analysis methods taught during the workshop can be applied to African wildlife species. Throughout the workshop, we used actual example data sets from African wildlife species.
20	Basic molecular biology and bioinformatics analysis	West Africa	University of Port Harcourt, Nigeria	Sample collection, basic molecular biology techniques and bioinformatics	The workshop consisted of a theoretical and practical session. The theoretical session introduced the fundamentals of molecular biology while the practical session provided hands-on training on sample collection including DNA extraction from plants (*Azadirachta indica* and *Portulaca olerace*a) using the cetyltrimethylammonium bromide (CTAB) method^157^, evaluation of the purity and concentration of genomic DNA through NanoDrop spectrophotometer, assessment of DNA quality using gel electrophoresis, and PCR amplification of the ribulose-1,5-bisphosphate carboxylase (Rubisco) gene from the extracted gDNA^158^. The hands-on training also included basic bioinformatics analysis, sequence alignment, and phylogenetic tree construction.
21	Basic molecular biology	West Africa	Inqaba Biotec West Africa, Nigeria	Molecular biology practical workshop: Bridging theory and application	The practical workshop focused on essential molecular biology techniques, including core laboratory skills, DNA extraction, and polymerase chain reaction (PCR) for amplifying the 16S rRNA gene from bacterial DNA. DNA fragments were visualized through gel electrophoresis, followed by sequencing the amplified DNA fragments and analyzing the sequencing data to integrate experimental procedures with data interpretation.
22	Basic Molecular Biology	West Africa	MyAfroDNA, Nigeria	Hands-on training on basic molecular biology techniques and bioinformatics	MyAfroDNA, in collaboration with AfricaBP, hosted a two-day workshop that provided participants with foundational laboratory skills, including pipetting, DNA extraction from plants, and nucleic acid quality analysis. Advanced sessions covered PCR, gel electrophoresis, and bioinformatics training on DNA sequencing, database navigation, BLAST searches, sequence annotation, and primer design. By integrating hands-on activities with computational tools, the workshop equipped attendees with essential skills for modern genetics/genomic research.
23	Sample collection and biobanking	Southern Africa	South African National Biodiversity Institute (SANBI)	Sample collection, processing and banking for use in genomic-level research	This workshop provided a comprehensive introduction to fauna and flora sample collection and biobanking for research and conservation. On Day 1, participants explored the value of biodiversity biobanks as well as an introduction to the Biodiversity Biobanks South Africa (BBSA) project^159^, and discussed ethics, permits, and compliance requirements, especially on animal species. Day 2 focused on sampling methodologies, including genetic and forensic sampling, taxon-specific techniques on birds, mammals and reptiles, and practical sessions on voucher photography and chain-of-custody sampling. Day 3 covered biodiversity data standards, BOLD database management, and plant sampling for DNA and herbarium collections.
24	Ethics, legal and social issues	Southern Africa	University of South Africa, South Africa	Ethics, Law, and Social Implications (ELSI) of genetic research for the African BioGenome Project	This workshop addressed the complexities of genomics research, particularly regarding indigenous knowledge and biopiracy. The workshop featured materials and hands-on exercises centered on themes such as prior informed consent, the ethical use of indigenous knowledge, and compliance with international frameworks like the Nagoya and Cartagena Protocols^160,161^. Participants explored equitable benefit-sharing strategies through activities guided by a practical workbook, including drafting ethical protocols, data transfer agreements, and benefit-sharing contracts. A key highlight was the San-Hoodia case study^162^, which illustrated ethical lapses in consent and indigenous knowledge exploitation. This case incited group discussions and role-playing exercises to develop ethical engagement strategies and fair commercialization practices. Participants also examined international frameworks governing access to genetic resources, addressing hypothetical challenges and proposing solutions to ensure compliance with both international and national laws. Due to logistical challenges, participants were unable to attend the workshop in-person. Instead, the course content was delivered via electronic emails, enabling participants to engage with the materials independently and at their own pace.

Several new hands-on practical workshop sites were added in 2024 and these include, but not limited to, IGH in Nigeria, Pwani University in Kenya, North-West University (NWU) and Stellenbosch University (SU) in South Africa, College of Computing at the University Mohammed VI Polytechnic (UM6P) in Morocco, and Higher Institute of Applied Biological Sciences of Tunis (ISSBAT) in Tunisia, while new hands-on practical workshop analysis types added in 2024 were simple sequence repeats (SSR) and transposable elements (TEs) in plants as well as genome editing using CRISPR/Cas9 technology (Table 1). For instance, Illumina’s hands-on practical workshops held at IGH, Nigeria, introduced participants to Illumina sequencing technology, in collaboration with their Africa-based partner, ISN Medical. This session focused on DNA extraction, PCR-free library preparations, sequencing and bioinformatics analysis of sequenced data, covering high-throughput sequencing platforms like the Illumina NextSeq 2000 and Illumina NovaSeq 6000 systems.

Similarly, the Oxford Nanopore Technology workshop, hosted at the University of Pretoria, South Africa, in collaboration with Distributed Platform in OMICS (DIPLOMICS), introduced participants to sequencing technologies with a focus on the PromethION flow cell system. Additionally, the MGI workshop, held at the Agricultural Research Council’s Biotechnology Platform, South Africa, demonstrated the full next-generation sequencing workflow using DNA Nanoballs Sequencing (DNBSEQ) technology, and finally, the Inqaba Biotec workshop in Nairobi, Kenya, provided participants with practical training in molecular biology and bioinformatics focused on DNA sequencing techniques, including DNA extraction from sponges and blood samples, PCR amplification, gel electrophoresis, and quality control assessment and sequencing data analysis.

The College of Computing at the University Mohammed VI Polytechnic (UM6P) in Morocco hosted a practical workshop on Simple Sequence Repeats and Transposable Elements in plants (Table 1), providing a comprehensive exploration of repetitive DNA sequences, their biological significance and the tools available for their identification and analysis. The workshop highlighted the importance of these tools for the progress of plant genome research. Additionally, the African Genome Center (AGC) at UM6P hosted a hands-on practical workshop, in partnership with Eppendorf and Thermo Fisher Scientific, which focused on genome editing using Clustered Regularly Interspaced Short Palindromic Repeats/CRISPR-associated protein 9 (CRISPR/Cas9) technology. CRISPR/Cas9 is one of the most revolutionary technologies in modern biology, enabling precise modifications of plant and animal genomes, with wide-ranging applications in research and agriculture^106^. The workshop also covered Basic Local Alignment Search Tool (BLAST) search and guide RNA (gRNA) design, both cloning-based and cloning-free editing techniques, and analysis of edited samples. In addition to the new themes, three new locations hosted practical workshops focused on genomic sequencing and bioinformatics, these include NWU and SU in South Africa and the ISSBAT in Tunisia. NWU’s practical workshop focused on Oxford Nanopore Technologies, covering library preparation, sequencing, and data analysis, SU’s Pathology Research Facility featured theoretical and practical sessions on metagenomic study design, DNA extraction and quality assessment, sequencing library preparation using the Oxford Nanopore MinION platform, and bioinformatics analysis while ISSBAT offered training on molecular biology techniques, metabarcoding, and microbial diversity analysis.

## Fellowships and infrastructural development

In addition to the AfricaBP Open Institute’s efforts to empower African researchers and promote collaborations through annual regional workshops in 2024, the AfricaBP Open Institute also launched or awarded training fellowships through multi-stakeholder partnerships as well as defined roadmap for the African digital sequence information (DSI) databank containing information relating to biodiversity. Here, we discuss the training contents for these fellowships (see El Allali et al.^107^ for details of the African DSI databank roadmap^107^):

### African Genome Center and AfricaBP Open Institute Joint Fellowship in Biodiversity Genomics and Bioinformatics

The AfricaBP Open Institute partnered with the African Genome Center (AGC) at the Mohammed VI Polytechnic University (UM6P) in Morocco to award an inaugural two-week residential and intensive hands-on Africa-based fellowship program for 10 African researchers^3^ in April 2024, which were selected from a pool of 545 applicants. The fellowship focused on the sequencing and analysis of the genome of *Vachellia gummifera*, a Moroccan endemic plant species of ecological and medical significance^108^. DNA extraction was followed by library preparation using the PCR-Free library kit to minimize biases typically associated with amplification-based methods^109^, and sequenced on the Illumina NextSeq 550 platform. The course content focused on building skills in high-throughput sequencing technologies, data quality control, and genome assembly. As a result, participants developed expertise in processing large genomic datasets and understanding species-specific genomic structures.

### African biodiversity fellowship for emerging genomics leaders

The AfricaBP Open Institute partnered with Carl R. Woese Institute for Genomic Biology (IGB) at the University of Illinois Urbana-Champaign, United States^3^ to award an inaugural 3 months leadership fellowship to 4 African researchers from Morocco, Egypt, South Africa, and Nigeria, respectively, out of a total of 66 applications. The fellowship involves two phases: A one month Africa phase (May–September 2024) which was hosted by the AGC in Morocco, the International Center of Agriculture Research in Dry Areas (ICARDA) in Egypt, Inqaba Biotechnical Industries in South Africa, and MyAfroDNA in Nigeria, respectively, and a residential 3-month international phase (September–December 2024) which was hosted and funded by the IGB.

At the AGC, the training focused on genomic data analysis of Moroccan sheep and QTL mapping to identify regions associated with specific traits across different breeds. This bioinformatics-based program utilized R software for statistical computing^110^ for genetic diversity analysis and Python for QTL mapping through SheepQTLdb database^111^. The aim was to investigate the genetic basis of key traits, with potential applications in breeding programs and livestock management. At ICARDA, the training focused on the pipeline for Genome-Wide Association Studies for crop improvement using the GAPIT tool^112^ in R studio^110^ and programming and scripting using Python and Bash and handling file formats such as VCF^113^. At Inqaba Biotechnical Industries, the training focused on the Linux command line, bash scripting, and South Africa’s Center for High-Performance Computing (CHPC) platform’s PBS scheduler, job submission, job monitoring, job arrays, and environment modules, as well as theoretical and practical insights into PacBio Revio. At MyAfroDNA, the training focused on DNA extraction, polymerase chain reaction, and Sanger sequencing processes.

This international phase focused on providing research exposure and advanced skill development, equipping African fellows with team science techniques applicable to genomics leadership in biodiversity research. At the IGB, each fellow was posted to one of the 15 multi-investigator, multidisciplinary Research Themes: Center for Genomic Diagnostics, Genomic Ecology of Global Change, and Biosystems Design, respectively.

Within the Center for Genomic Diagnostics, fellows: (a) explored the use of nanomaterials for targeted drug delivery, imaging, and therapeutic applications, novel drug delivery systems, such as nanocarriers and smart materials, that can more efficiently deliver cancer therapies while reducing side effects, (b) executed a project on the use of advanced diagnostic tools and biosensors for disease detection and monitoring, (c) performed analysis using scanning electron microscopy for the examination of material compositions and surface structures at the microscale, transmission electron microscopy for the high-resolution characterization of the size, shape, and morphology of nanomaterials, and Immunofluorescence and the theory, use, and interpretation of enzyme-linked immunosorbent assay data, (d) introduced to mammalian cell culture, flow cytometry, confocal microscopy, fluorescence microscopy, and cell viability tests such as MTT assays to identify the best materials for targeted drug administration, and (e) examined the hotspot point mutations in acute myeloid leukemia (AML) utilizing the computational biology expertise and knowledge they acquired during the program’s African phase.

Within the Genomic Ecology of Global Change Theme, one of the fellows was assigned to the photosynthesis engineering laboratory, which focuses on enhancing photosynthesis in crops and algae by studying molecular mechanisms and identifying components that reveal plant responses to external and internal cues. The fellow carried out a project on the dual nature of plant genome promoters, tested their expression levels in plants, and bioinformatically identified native bidirectional promoters^114^, which are 1000 bp intergenic regions between head-to-head gene pairs. This research was performed in the model plant *Arabidopsis thaliana* using R scripting and bash programming. Candidate bidirectional pairs were filtered based on the proximity between gene pairs and their correlation to identify potential bidirectional promoters. After filtration, gene pairs were categorized into positive correlation (genes that tend to increase expression and together) and negative correlation (genes that tend to decrease expression together)^115^ while the bidirectional promoters assigned to the positively correlated genes were programmed for further screening via transient transformation into other model plants.

Finally, one of the fellows was assigned to The Biosystems Design Theme, which focuses on developing and applying synthetic biology, machine learning, and laboratory automation tools to address society’s most daunting challenges in human health and energy and investigating the fundamental aspects of enzyme catalysis, cell metabolism, and gene regulation^116^. The fellow’s project focused on developing a new-to-nature hydroaminoalkylation reaction for the synthesis of chiral amines, which serve as useful building blocks to pharmaceuticals, agrochemicals, and specialty chemicals by synergizing photocatalysis and biocatalysis and by using directed evolution. Some of the techniques employed in the course of the project include protein engineering, high-performance liquid chromatography, and identification of biosynthetic gene clusters (BGCs) using resources such as antiSMASH^117^.

### African plant genome assembly and annotation fellowship

The AfricaBP Open Institute partnered with the International Institute of Tropical Agriculture (IITA), Nigeria, and Inqaba Biotec, West Africa, to launch the inaugural edition of the African plant genome assembly and annotation fellowship in June 2024, which will feature a hybrid format fellowship combining an 8-week virtual phase starting in Q1 2025 and a 10-day on-site intensive training at the IITA headquarters in Ibadan, Nigeria, scheduled for April 2025. A total of 10 fellows have been selected from 261 applicants, with notifications scheduled for first quarter of 2025. The virtual phase is designed to equip participants with advanced knowledge in genome biology, PacBio HiFi and Omni-C sequence reads, methods for data quality control, assembling and assessing long- and short-reads, and estimating genome assembly completeness. It will also include training in gene prediction and functional annotation techniques, including annotating gene sets with associated functional information.

## **Conclusion, recommendations, and future directions**

The AfricaBP Open Institute is making intentional progress in addressing AfricaBP’s Grand Challenges, focusing, in 2024, on using biodiversity genomics and bioinformatics to support Africa’s bioeconomy. By combining advanced technologies with local strategies, AfricaBP has expanded its understanding of bioeconomy frameworks and identified sustainable pathways to address Africa’s biodiversity challenges. One key effort is providing a platform for national networks of scientists to expand local sequencing infrastructures and capabilities. The proposed 1000 Moroccan Genome Project case study demonstrates how investments in genomics can deliver significant returns, with a 3.29 Benefit–Cost ratio and $28 million net present value over10 years. Establishing local sequencing hubs will reduce foreign dependency, create skilled jobs, lower costs, and accelerate innovation, strengthening Africa’s competitiveness in the global genomics landscape. Equally important is the focus on high-impact agricultural applications. Prioritizing the development of drought-resistant crops and disease-resilient livestock could address over 53% of Africa’s projected economic output tied to agriculture, while also enhancing food security and positioning African nations as leaders in sustainable agricultural innovation.

Capacity building and strengthening remains a central pillar of AfricaBP’s strategy. Through specialized curricula, research programs, and inter-Africa and inter-continental collaborations, the AfricaBP Open Institute is cultivating a skilled genomics and bioinformatics workforce capable of driving innovation in agriculture and biodiversity conservation. Strengthened public–private partnerships, supported by fiscal incentives, streamlined regulations, and transparent frameworks, will attract investment, accelerate technology transfer, and stimulate market growth for genomics-based products. Additionally, AfricaBP’s roadmap for a transformative digital sequence information (DSI) database marks a critical step forward. Directing sequencing efforts toward high-priority species in biodiversity-rich areas will protect Africa’s genetic resources, enhance climate resilience, and support ecosystems critical to sustainable economic gains. To ensure lasting impact, AfricaBP is advocating for robust data policies aligned with national genetics resources and digital sequence information framework^98,107^. These policies aim to safeguard genomic information, preserve indigenous knowledge, and ensure equitable benefit-sharing, creating a foundation that aligns genomics research with broader regional priorities and further accelerates Africa’s collective bioeconomy potential.

## Supplementary information

Supplementary Information

## References

[1] Ebenezer, T. E. et al. Africa: sequence 100,000 species to safeguard biodiversity. *Nature* **603**, 388–392 (2022). 35292740 10.1038/d41586-022-00712-4

[2] Sharaf, A. et al. Bridging the gap in African biodiversity genomics and bioinformatics. *Nat. Biotechnol.* **41**, 1348–1354 (2023). 37699986 10.1038/s41587-023-01933-2

[3] Sharaf, A. et al. Establishing African genomics and bioinformatics programs through annual regional workshops. *Nat. Genet.* **56**, 1556–1565 (2024). 38977855 10.1038/s41588-024-01807-6

[4] Mougenot, B. & Doussoulin, J.-P. Conceptual evolution of the bioeconomy: a bibliometric analysis. *Environ. Dev. Sustain.* **24**, 1031–1047 (2022). 33967598 10.1007/s10668-021-01481-2 PMC8096632

[5] Global Bioeconomy Summit. *Communiqué Global Bioeconomy Summit 2018: Innovation in the Global Bioeconomy for Sustainable and Inclusive Transformation and Wellbeing*(Global Bioeconomy Summit, accessed 12 Jan 2025); https://www.biooekonomierat.de/media/pdf/archiv/international-gbs-2018-communique.pdf (2018).

[6] Patermann, C. & Aguilar, A. A bioeconomy for the next decade. *EFB Bioecon. J.* **1**, 100005 (2021).

[7] Nature Finance & Getúlio Vargas Foundation. *Preliminary Stocktake of G20 Strategies and Practices: a Contribution to the Brazilian G20 Presidency’s Global Initiative on Bioeconomy*(Nature Finance & Getúlio Vargas Foundation, accessed 13 Jan 2025). https://www.naturefinance.net/wp-content/uploads/2024/05/ENG-TheGlobalBioeconomy_FINAL.pdf (2025).

[8] World Bioeconomy Forum. *A Status of the Global Bioeconomy* (World Bioeconomy Forum, accessed 28 Dec 2024) https://wcbef.com/tuote/a-status-of-the-global-bioeconomy/ (2024).

[9] El-Chichakli, B., Von Braun, J., Lang, C., Barben, D. & Philp, J. Policy: five cornerstones of a global bioeconomy. *Nature* **535**, 221–223 (2016). 27411618 10.1038/535221a

[10] Gould, H., Kelleher, L. & O’Neill, E. Trends and policy in bioeconomy literature: a bibliometric review. *EFB Bioecon. J.* **3**, 100047 (2023).

[11] Jiménez-Sánchez, G. & Philp, J. Omics and the bioeconomy: applications of genomics hold great potential for a future bio-based economy and sustainable development. *EMBO Rep.* **16**, 17–20 (2015). 25476709 10.15252/embr.201439409 PMC4304725

[12] Chitaka, T. Y. & Schenck, C. Developing country imperatives in the circular bioeconomy: a review of the South African case. *Environ. Dev.* **45**, 100812 (2023).

[13] Food and Agriculture Organization of the United Nations. *Assessing the Contribution of Bioeconomy to Countries’ Economy: a Brief Review of National Frameworks* (Food and Agriculture Organization of the United Nations, accessed 12 Jan 2025) https://www.fao.org/3/I9580EN/i9580en.pdf (2018).

[14] Morris, E. J. Moving Africa towards a knowledge-based bio-economy. In *Biotechnology in Africa* Vol. 7 (eds Wambugu, F. & Kamanga, D.) 69–93 (Springer International Publishing, Cham, 2014).

[15] Mulder, N. J. et al. The development of computational biology in South Africa: successes achieved and lessons learnt. *PLoS Comput. Biol.* **12**, e1004395 (2016). 26845152 10.1371/journal.pcbi.1004395 PMC4742231

[16] Pauwels, E. & Tilmes, K. The Artificial Intelligence—Biotech Revolution in Africa. In *Africa–Europe Cooperation and Digital Transformation* (eds. Daniels, C., Erforth, B. & Teevan, C.) 50–65 10.4324/9781003274322 (Routledge, London, 2022).

[17] Aidoo, R., Kwofie, E. M., Glatzel, K. & Ecuru, J. Bioeconomy: a path to African food system transformation. In *African Food Systems Transformation and the Post-Malabo Agenda* (eds Ulimwengu, J. M., Kwofie, E. M. & Collins, J.) Ch. 10, 173–188 (AKADEMIYA2063; International Food Policy Research Institute (IFPRI), Kigali, Rwanda; Washington, DC) https://agris.fao.org/search/en/providers/122566/records/6582fba8ebe24a6dee898d70 (2023).

[18] Feleke, S., Cole, S. M., Sekabira, H., Djouaka, R. & Manyong, V. Circular bioeconomy research for development in sub-Saharan Africa: innovations, gaps, and actions. *Sustainability* **13**, 1926 (2021).

[19] Oguntuase, O. J. Bioeconomy for sustainable development in Africa—state of production determinants and future directions. *Stud. Ekonom. Reg.* **13**, 1–14 (2020).

[20] Oguntuase, O. J. & Adu, O. B. Bioeconomy as climate action: how ready are African countries? In *African Handbook of Climate Change Adaptation* (eds. Leal Filho, W., Oguge, N., Ayal, D., Adeleke, L. & Da Silva, I.) 1–15 (Springer International Publishing, Cham 2021).

[21] MarketsandMarkets. *Genomics Market Growth, Drivers, And Opportunities* https://www.marketsandmarkets.com/Market-Reports/genomics-market-613.html (MarketsandMarkets, 2024).

[22] Walsh, K. & Spazzoli, R. *Assessing the Economic Impact of the South African Beef Genomics Programme* https://www.novaeconomics.co.za/our-work/705-2 (2018).

[23] Omotoso, O. E. et al. Bridging the genomic data gap in Africa: implications for global disease burdens. *Global Health* **18**, 103 (2022). 36494695 10.1186/s12992-022-00898-2 PMC9733397

[24] Carstens, N., Glanzmann, B., Kinnear, C. & Uren, C. Next-generation sequencing technologies: implementation in developing countries. In *Population Genomics in the Developing World* (eds Patrinos, G.P., Möller, M. & Uren, C.) 83–90 10.1016/B978-0-443-18546-5.00005-X (Academic Press, 2025).

[25] Wudil, A. H., Usman, M., Rosak-Szyrocka, J., Pilař, L. & Boye, M. Reversing years for global food security: a review of the food security situation in Sub-Saharan Africa (SSA). *IJERPH* **19**, 14836 (2022). 36429555 10.3390/ijerph192214836 PMC9690952

[26] Adepoju, P. Africa’s future depends on government-funded R&D. *Nat. Africa*10.1038/D44148-022-00134-4 (2022)

[27] Okoye, K., Nganji, J. T., Escamilla, J., Fung, J. M. & Hosseini, S. Impact of global government investment on education and research development: a comparative analysis and demystifying the science, technology, innovation, and education conundrum. *Global Transit.* **4**, 11–27 (2022).

[28] Caelers, D. & Okoth, D. Research funding in Africa: navigating sustainability and shifting perspectives. *Nat Africa*10.1038/D44148-023-00360-4 (2023)

[29] Leontief, W. W. Quantitative input and output relations in the economic systems of the United States. *Rev. Econ. Stat.* **18**, 105 (1936).

[30] Neugarten, R. A. et al. Rapid assessment of ecosystem service co-benefits of biodiversity priority areas in Madagascar. *PLoS ONE* **11**, e0168575 (2016). 28006005 10.1371/journal.pone.0168575 PMC5179119

[31] Otieno, J. O. Challenges and current strategies in biodiversity conservation in Kenya: a review. *OALib* **10**, 1–17 (2023).

[32] Adebamowo, S. N. et al. Implementation of genomics research in Africa: challenges and recommendations. *Global Health Action* **11**, 1419033 (2018). 29336236 10.1080/16549716.2017.1419033 PMC5769805

[33] Oboh, M. A. et al. Translation of genomic epidemiology of infectious pathogens: enhancing African genomics hubs for outbreaks. *Int. J. Infect. Dis.* **99**, 449–451 (2020). 32800861 10.1016/j.ijid.2020.08.027 PMC7423511

[34] Acevedo, M. et al. A scoping review of adoption of climate-resilient crops by small-scale producers in low- and middle-income countries. *Nat. Plants* **6**, 1231–1241 (2020). 33051616 10.1038/s41477-020-00783-z PMC7553851

[35] Bond, M. R. & Scott, D. Digital biopiracy and the (dis)assembling of the Nagoya Protocol. *Geoforum* **117**, 24–32 (2020). 33041359 10.1016/j.geoforum.2020.09.001 PMC7536632

[36] Xu, H. Does government support affect private partners’ profitability in public–private partnerships? Evidence from China. *Humanit. Soc. Sci. Commun.* **10**, 1–10 (2023).

[37] Proestou, M., Schulz, N. & Feindt, P. H. A global analysis of bioeconomy visions in governmental bioeconomy strategies. *Ambio* **53**, 376–388 (2024). 38151615 10.1007/s13280-023-01958-6 PMC10837399

[38] Bio and Emerging Technology Institute. *Ethiopian Bioeconomy Strategy*. MinT. https://www.betin.gov.et/wp-content/uploads/2024/11/Draft-Ethiopian-Bioeconomy-Strategy.pdf (Bio and Emerging Technology Institute, 2024).

[39] Malabo Montpellier Panel. *ADOPT: Policy Innovations to Unlock Climate Finance for Resilient Food Systems in Africa* (Malabo Montpellier Panel, accessed 13 Jan 2025) https://reliefweb.int/report/world/adopt-policy-innovations-unlock-climate-finance-resilient-food-systems-africa (2022).

[40] Glatzel, K. et al. *Bioeconomy Pathways: Experience from Africa, Asia, and Latin America* https://hdl.handle.net/10568/155083 (2024).

[41] Adeleke, B., Robertson-Andersson, D., Moodley, G. & Taylor, S. Aquaculture in Africa: a comparative review of Egypt, Nigeria, and Uganda Vis-À-Vis South Africa. *Rev. Fish. Sci. Aquacult.* **29**, 167–197 (2021).

[42] Matekenya, W. & Ncwadi, R. Impact of financing aquaculture on selected economic indicators in South Africa: cointegration approach. *J. Appl. Aquacult.* **36**, 193–214 (2022).

[43] Tshilate, T. S., Ishengoma, E. & Rhode, C. A first annotated genome sequence for Haliotis midae with genomic insights into abalone evolution and traits of economic importance. *Mar. Genom.* **70**, 101044 (2023). 10.1016/j.margen.2023.101044 37196472

[44] Tshilate, T. S., Ishengoma, E. & Rhode, C. Construction of a high-density linkage map and QTL detection for growth traits in South African abalone (Haliotis midae). *Animal Genet.* **55**, 744–760 (2024). 38945682 10.1111/age.13462

[45] Henriques, R. et al. Spatio-temporal genetic structure and the effects of long-term fishing in two partially sympatric offshore demersal fishes. *Mol. Ecol.* **25**, 5843–5861 (2016). 27862532 10.1111/mec.13890

[46] Forde, S. et al. Management and conservation implications of cryptic population substructure for two commercially exploited fishes (*Merluccius* spp.) in southern Africa. *Mol. Ecol. Resour.*10.1111/1755-0998.13820 (2023). 10.1111/1755-0998.13820 PMC12142719 37291747

[47] Darias, M. J., Brink-Hull, M., Bach, P. & Macey, B. M. *Accelerating Transformation in Aquatic Food Systems Through Holistic And Inclusive Innovation: Building And Strengthening Innovation Ecosystems* https://ird.hal.science/ird-04832297 (2024).

[48] O’Donohoe, P. et al. *Five Integrated Multitrophic Aquaculture Laboratories, One Goal—Resilient Aquaculture in the Face of Emerging Threats*. ASTRAL Project https://www.researchgate.net/profile/Tomas-Chalde/publication/377804841_FIVE_INTEGRATED_MULTITROPHIC_AQUACULTURE_LABORATORIES_ONE_GOAL_-RESILIENT_AQUACULTURE_IN_THE_FACE_OF_EMERGING_THREATS_ASTRAL_PROJECT/links/65b8ff3a79007454974f201d/FIVE-INTEGRATED-MULTITROPHIC-AQUACULTURE-LABORATORIES-ONE-GOAL-RESILIENT-AQUACULTURE-IN-THE-FACE-OF-EMERGING-THREATS-ASTRAL-PROJECT.pdf (2024).

[49] East African Community. *The East African Regional Bioeconomy Strategy* 2021/22–2031/32 (East African Community (EAC), 2022).

[50] Mensah, A. M. & Gordon, C. Strategic partnerships between universities and non-academic institutions for sustainability and innovation: insights from the University of Ghana. In *Sustainability Challenges in Sub-Saharan Africa I: Science for Sustainable Societies.* 245–278 10.1007/978-981-15-4458-3_8 (Springer, Singapore 2020).

[51] Ghildiyal, K. et al. Genomic insights into the conservation of wild and domestic animal diversity: a review. *Gene* **886**, 147719 (2023). 37597708 10.1016/j.gene.2023.147719

[52] Theissinger, K. et al. How genomics can help biodiversity conservation. *Trends Genet.* **39**, 545–559 (2023). 36801111 10.1016/j.tig.2023.01.005

[53] Rhie, A. et al. Towards complete and error-free genome assemblies of all vertebrate species. *Nature* **592**, 737–746 (2021). 33911273 10.1038/s41586-021-03451-0 PMC8081667

[54] Rege, J. E. O. The state of African cattle genetic resources I. Classification framework and identification of threatened and extinct breeds. *Animal Genet. Resour. Inf.* **25**, 1–25 (1999).

[55] Rege, J. E. O. & Tawah, C. L. The state of African cattle genetic resources II. Geographical distribution, characteristics and uses of present-day breeds and strains. *Animal Genet. Resour. Inf.* **26**, 1–25 (1999).

[56] Houaga, I. et al. *High Quality Genome Assemblies of African Cattle Breeds using PacBio HiFi Sequencing*10.1101/2025.04.17.649430 (2025). 10.1038/s41597-026-07589-2 PMC13562511 42251053

[57] Friedrich, J., Liu, S., Fang, L., Prendergast, J. & Wiener, P. Insights into trait-association of selection signatures and adaptive eQTL in indigenous African cattle. *BMC Genom.* **25**, 981 (2024). 10.1186/s12864-024-10852-8 PMC11490109 39425030

[58] Sinha, P. et al. Genomics and breeding innovations for enhancing genetic gain for climate resilience and nutrition traits. *Theor. Appl. Genet.* **134**, 1829–1843 (2021). 34014373 10.1007/s00122-021-03847-6 PMC8205890

[59] Ghavi Hossein-Zadeh, N. An overview of recent technological developments in bovine genomics. *Vet. Animal Sci.* **25**, 100382 (2024). 10.1016/j.vas.2024.100382 PMC11334705 39166173

[60] Kidambasi, K. O., Masiga, D. K., Villinger, J., Carrington, M. & Bargul, J. L. Detection of blood pathogens in camels and their associated ectoparasitic camel biting keds, *Hippobosca camelina*: the potential application of keds in xenodiagnosis of camel haemopathogens. *AAS Open Res.* **2**, 164 (2020). 32510036 10.12688/aasopenres.13021.1 PMC7243205

[61] Adeoba, M., Tesfamichael, S. G. & Yessoufou, K. Preserving the tree of life of the fish family Cyprinidae in Africa in the face of the ongoing extinction crisis. *Genome* **62**, 170–182 (2019). 30865849 10.1139/gen-2018-0023

[62] Doungous, O. et al. Cassava mosaic disease and its whitefly vector in Cameroon: incidence, severity and whitefly numbers from field surveys. *Crop Prot.* **158**, 106017 (2022). 35923211 10.1016/j.cropro.2022.106017 PMC9168542

[63] Monteiro, F. et al. Tracking cashew economically important diseases in the West African region using metagenomics. *Front. Plant Sci*. **6**, 10.3389/fpls.2015.00482 (2015) 10.3389/fpls.2015.00482 PMC4485029 26175748

[64] Oginyi, J. C., Chukwu, S. C., Paul, K. U. & Mkpuma, K. C. Genetic diversity and stability analysis based on agro-morphological traits among rice genotypes developed through marker-assisted backcrossing. *Int. J. Sci. Rep.* **10**, 148–155 (2024).

[65] El Faqer, A., Rabeh, K., Alami, M., Filali-Maltouf, A. & Belkadi, B. In silico identification and characterization of fatty acid desaturase (FAD) genes in *Argania spinosa* L. skeels: implications for oil quality and abiotic stress. *Bioinform. Biol. Insights* **18**, 11779322241248908 (2024). 38711943 10.1177/11779322241248908 PMC11072076

[66] Elias, M. et al. Multi-locus genome-wide association study reveal genomic regions underlying root system architecture traits in Ethiopian sorghum germplasm. *Plant Genome* **17**, e20436 (2024). 38361379 10.1002/tpg2.20436 PMC12807215

[67] Habimana, V. et al. Genes and models for estimating genetic parameters for heat tolerance in dairy cattle. *Front. Genet.* **14**, 10.3389/fgene.2023.1127175 (2023). 10.3389/fgene.2023.1127175 PMC10009153 36923799

[68] Ouhrouch, A. et al. Genomic uniqueness of local sheep breeds from Morocco. *Front. Genet*. **12**, 10.3389/fgene.2021.723599 (2021). 10.3389/fgene.2021.723599 PMC8675355 34925440

[69] Kusza, S., Badaoui, B. & Wanjala, G. Insights into the genomic homogeneity of Moroccan indigenous sheep breeds though the lens of runs of homozygosity. *Sci. Rep.* **14**, 16515 (2024). 39019985 10.1038/s41598-024-67558-w PMC11255268

[70] Benjelloun, B. et al. An evaluation of sequencing coverage and genotyping strategies to assess neutral and adaptive diversity. *Mol. Ecol. Resour.* **19**, 1497–1515 (2019). 31359622 10.1111/1755-0998.13070 PMC7115901

[71] Simma, S. et al. Diversité du génome entier et dynamique démographique de la race ovine D’man. *African Mediterr. Agric. J.—Al Awamia* 123–141. 10.34874/IMIST.PRSM/afrimed-i138.39134 (2023).

[72] Chafai, N., Hayah, I., Houaga, I. & Badaoui, B. A review of machine learning models applied to genomic prediction in animal breeding. *Front. Genet.* **14**, 1150596 (2023). 37745853 10.3389/fgene.2023.1150596 PMC10516561

[73] Ishengoma, E. Vertebrate genomics and adaptation—status and prospects in Africa. *Mol. Ecol.* **32**, 3368–3381 (2023). 36946911 10.1111/mec.16936

[74] Liu, X. et al. Introgression and disruption of migration routes have shaped the genetic integrity of wildebeest populations. *Nat. Commun.* **15**, 2921 (2024). 38609362 10.1038/s41467-024-47015-y PMC11014984

[75] Talenti, A. et al. Continent-wide genomic analysis of the African buffalo (*Syncerus caffer*). *Commun. Biol.* **7**, 792 (2024). 38951693 10.1038/s42003-024-06481-2 PMC11217449

[76] Rattray, R. D., Stewart, R. D., Niemann, H. J., Olaniyan, O. D. & van der Bank, M. Leafing through genetic barcodes: an assessment of 14 years of plant DNA barcoding in South Africa. *S. Afr. J. Bot.* **172**, 474–487 (2024).

[77] Stewart, R. D., Bard, N., Van Der Bank, M. & Davies, T. J. *Leveraging Machine Learning and Citizen Science Data to Describe Flowering Phenology Across South Africa*. 10.1101/2023.12.21.572952 (2023).

[78] Volpato, A. et al. Using Malaise traps to assess aculeate Hymenoptera associated with farmland linear habitats across a range of farming intensities. *Insect Conserv. Divers.* **13**, 229–238 (2020).

[79] Folarin, O. A. et al. Ebola virus epidemiology and evolution in Nigeria. *J. Infect .Dis.* **214**, S102–S109 (2016). 27377746 10.1093/infdis/jiw190 PMC5050462

[80] Wilkinson, E. et al. A year of genomic surveillance reveals how the SARS-CoV-2 pandemic unfolded in Africa. *Science* **374**, 423–431 (2021). 34672751 10.1126/science.abj4336 PMC7613315

[81] Kotliar, D. et al. Genome-wide association study identifies human genetic variants associated with fatal outcome from Lassa fever. *Nat. Microbiol* **9**, 751–762 (2024). 38326571 10.1038/s41564-023-01589-3 PMC10914620

[82] Hending, D. Cryptic species conservation: a review. *Biol. Rev.* **100**, 258–274 (2025). 39234845 10.1111/brv.13139 PMC11718601

[83] Bertola, L. D. et al. Giraffe lineages are shaped by major ancient admixture events. *Curr. Biol.* **34**, 1576–1586.e5 (2024). 38479386 10.1016/j.cub.2024.02.051

[84] Calcino, A. et al. Harnessing genomic technologies for one health solutions in the tropics. *Global Health* **20**, 78 (2024). 39543642 10.1186/s12992-024-01083-3 PMC11566161

[85] Marshall, K. et al. Livestock genomics for developing countries—African examples in practice. *Front. Genet.* **10**, 297 (2019). 31105735 10.3389/fgene.2019.00297 PMC6491883

[86] Vilaça, S. T. et al. Leveraging genomes to support conservation and bioeconomy policies in a megadiverse country. *Cell Genom.* **4**, 100678 (2024). 39423822 10.1016/j.xgen.2024.100678 PMC11605686

[87] Mittermeier, R. A., Mittermeier, C. G. & Gil, P. R. *Megadiversity: Earth’s Biologically Wealthiest Nations* (CEMEX) https://books.google.co.ma/books?id=wvObQAAACAAJ (1997).

[88] Humphrey, S. *Africa Ecological Footprint Report: Green Infrastructure for Africa’s Security* (WWF International and African Development Bank, 2012).

[89] Karani, P., Failler, P., Gilau, A. M., Ndende, M. & Diop, S. T. Africa blue economy strategies integrated in planning to achieve sustainable development at National and Regional Economic Communities (RECs). *J. Sustain. Res*. 10.20900/jsr20220011 (2022).

[90] South African National Biodiversity Institute (SANBI). *National Biodiversity Assessment 2018: The Status of South Africa’s Ecosystems and Biodiversity.* Synthesis Report (South African National Biodiversity Institute, Pretoria, 2018).

[91] Nwosu, O. K. & Bello, S. Overview of the biosafety system in Nigeria. *OBC-WPRS**Bull*. **163**, 79–84 http://iobc-wprs.org/product/overview-of-the-biosafety-system-in-nigeria (2023).

[92] UNEP Convention on Biological Diversity. in *Conference of the Parties to the Adopted Decision 15/4, Kunming-Montreal Global Biodiversity Framework. Fifteenth Meeting—Part II*, Montreal, Canada, 7–19 https://www.cbd.int/doc/decisions/cop-15/cop-15-dec-04-en.pdf (UNEP Convention on Biological Diversity, 2022).

[93] International Union for Conservation of Nature. *Kunming-Montreal Global Biodiversity Framework*. Issues Brief (International Union for Conservation of Nature, 2024).

[94] GBIF. *Scoping Study Explores a Global Nature-related Public Data Facility* (GBIF, 2023).

[95] GBIF. *Nigeria Rejoins GBIF as Voting Participant* (GBIF, 2023).

[96] Parker-Allie, F., Gibbons, M. J. & Harebottle, D. M. A conceptual approach to developing biodiversity informatics as a field of science in South Africa. *Front. Ecol. Evol.* **11**, 1107212 (2023).

[97] Rogalla Von Bieberstein, K. et al. Improving collaboration in the implementation of global biodiversity conventions. *Conserv. Biol.* **33**, 821–831 (2019). 30461056 10.1111/cobi.13252

[98] Katee, S. M. et al. *African Biodiversity Genomics in the Era of the Kunming-Montreal Global Biodiversity Framework: Ethical, Legal, and Social Perspectives*10.31219/OSF.IO/2XQ5E (2024).

[99] Kurth, T., Wübbels, G., Portafaix, A., Meyer zum Felde, A. & Zielcke, S. *The Biodiversity Crisis is a Business Crisis (Boston Consulting Group)* https://web-assets.bcg.com/fb/5e/74af5531468e9c1d4dd5c9fc0bd7/bcg-the-biodiversity-crisis-is-a-business-crisis-mar-2021-rr.pdf (2021).

[100] Poquérusse, J. et al. Assessing contemporary Arctic habitat availability for a woolly mammoth proxy. *Sci. Rep.* **14**, 9804 (2024). 38684726 10.1038/s41598-024-60442-7 PMC11058768

[101] Seddon, P. J., Moehrenschlager, A. & Ewen, J. Reintroducing resurrected species: Selecting de-extinction candidates. *Trends Ecol. Evol.* **29**, 140–147 (2014). 24513302 10.1016/j.tree.2014.01.007

[102] IPBES. *Global Assessment Report on Biodiversity And Ecosystem Services of the Intergovernmental Science-Policy Platform on Biodiversity and Ecosystem Services (Version 1)*. 10.5281/ZENODO.3831673 (IPBES, 2019).

[103] Newman, D. J. & Cragg, G. M. Natural products as sources of new drugs over the nearly four decades from 01/1981 to 09/2019. *J. Nat. Prod.* **83**, 770–803 (2020). 32162523 10.1021/acs.jnatprod.9b01285

[104] Adaji, A. E. & Abdulrauf, L. A. Intellectual property issues for open science practices in genomic-related health research and innovation in Africa. *J. Law Biosci.* **11**, lsae026 (2024). 39691103 10.1093/jlb/lsae026 PMC11649944

[105] Saeed, U. F., Kamil, R. & Wiredu, I. The roles of ICT and governance quality in the finance-growth nexus of developing countries: a dynamic GMM approach. *Cogent Econ. Financ*. **13**, 10.1080/23322039.2024.2448228 (2025).

[106] Bortesi, L. et al. Patterns of CRISPR/Cas9 activity in plants, animals and microbes. *Plant Biotechnol. J.* **14**, 2203–2216 (2016). 27614091 10.1111/pbi.12634 PMC5103219

[107] El Allali, A. et al. *Enabling Africa’s implementation of the Kunming-Montreal Global Biodiversity Framework through the African Digital Sequence Information Data Bank*. 10.31219/osf.io/anbwv (2024).

[108] Lahdachi, F. Z., Nassiri, L., Ibijbijen, J. & Mokhtari, F. Aperçu sur les acacias spontanés et introduits au Maroc. *Eur. Sci. J*. **11**, 1857–7881 retrieved from https://eujournal.org/index.php/esj/article/view/6071 (2015).

[109] Aird, D. et al. Analyzing and minimizing PCR amplification bias in Illumina sequencing libraries. *Genome Biol.* **12**, R18 (2011). 21338519 10.1186/gb-2011-12-2-r18 PMC3188800

[110] R Core Team. *R: A Language and Environment For Statistical Computing* https://www.R-project.org/ (R Core Team, 2020).

[111] Hu, Z. L., Park, C. A. & Reecy, J. M. Bringing the Animal QTLdb and CorrDB into the future: meeting new challenges and providing updated services. *Nucleic Acids Res.* **50**, D956–D961 (2022). 34850103 10.1093/nar/gkab1116 PMC8728226

[112] Lipka, A. E. et al. GAPIT: genome association and prediction integrated tool. *Bioinformatics* **28**, 2397–2399 (2012). 22796960 10.1093/bioinformatics/bts444

[113] Danecek, P. et al. The variant call format and VCFtools. *Bioinformatics* **27**, 2156–2158 (2011). 21653522 10.1093/bioinformatics/btr330 PMC3137218

[114] Wang, Z. et al. A WRKY transcription factor participates in dehydration tolerance in *Boea hygrometrica* by binding to the W-box elements of the galactinol synthase (BhGolS1) promoter. *Planta* **230**, 1155–1166 (2009). 19760263 10.1007/s00425-009-1014-3

[115] Lukowski, S. W. et al. Genetic correlations reveal the shared genetic architecture of transcription in human peripheral blood. *Nat. Commun.* **8**, 483 (2017). 28883458 10.1038/s41467-017-00473-z PMC5589780

[116] Volk, M. J. et al. Biosystems design by machine learning. *ACS Synth. Biol.* **9**, 1514–1533 (2020). 32485108 10.1021/acssynbio.0c00129

[117] Blin, K. et al. antiSMASH 5.0: updates to the secondary metabolite genome mining pipeline. *Nucleic Acids Res.* **47**, W81–W87 (2019). 31032519 10.1093/nar/gkz310 PMC6602434

[118] African BioGenome Project (AfricaBP). *Acknowledgements for the AfricaBP Open Institute Regional Workshops 2024*. https://osf.io/t78xq 10.17605/OSF.IO/2BQ7C (African BioGenome Project (AfricaBP), 2025).

[119] QGIS Development Team. *QGIS Geographic Information System. Version 3.x* (QGIS Development Team, 2024).

[120] Lex, A., Gehlenborg, N., Strobelt, H., Vuillemot, R. & Pfister, H. UpSet: Visualization of Intersecting Sets. *IEEE Trans. Visual. Comput. Graph.* **20**, 1983–1992 (2014). 10.1109/TVCG.2014.2346248 PMC4720993 26356912

[121] SAS Institute Inc. JMP®, Version 17.0 (SAS Institute Inc., Cary, NC, 1989–2023).

[122] 1KSA. *1KSA Decoding South Africa’s Biodiversity|Genomics* (1KSA, 2025).

[123] Scarano, C., et al. Antibiotic resistance of *Vibrio* species isolated from *Sparus aurata* reared in Italian mariculture. *New Microbiol.* **37**, 329–337 (2014). 25180847

[124] Tamura, K., Stecher, G. & Kumar, S. MEGA11: molecular evolutionary genetics analysis version 11. *Mol. Biol. Evol.* **38**, 3022–3027 (2021). 33892491 10.1093/molbev/msab120 PMC8233496

[125] Kekre, M. & Pascoe, B. *C-SOP-201: Genomic DNA Quantification Using a Qubit Fluorometer*10.17504/PROTOCOLS.IO.KQDG3XB47G25/V1 (2023).

[126] Li, D., Liu, C.-M., Luo, R., Sadakane, K. & Lam, T.-W. MEGAHIT: an ultra-fast single-node solution for large and complex metagenomics assembly via succinct *de Bruijn* graph. *Bioinformatics* **31**, 1674–1676 (2015). 25609793 10.1093/bioinformatics/btv033

[127] Flynn, J. M. et al. RepeatModeler2 for automated genomic discovery of transposable element families. *Proc. Natl Acad. Sci. USA* **117**, 9451–9457 (2020). 32300014 10.1073/pnas.1921046117 PMC7196820

[128] Smit, A. F. A., Hubley, R. & Green, P. *RepeatMasker* http://repeatmasker.org (2024).

[129] Mokhtar, M. M., Alsamman, A. M. & El Allali, A. MegaSSR: a web server for large scale microsatellite identification, classification, and marker development. *Front. Plant Sci.* **14**, 1219055 (2023). 38162302 10.3389/fpls.2023.1219055 PMC10757629

[130] Ou, S. et al. Benchmarking transposable element annotation methods for creation of a streamlined, comprehensive pipeline. *Genome Biol.* **20**(1), 1–18 (2019). 31843001 10.1186/s13059-019-1905-y PMC6913007

[131] Mokhtar, M. M. & El Allali, A. MegaLTR: a web server and standalone pipeline for detecting and annotating LTR-retrotransposons in plant genomes. *Front. Plant Sci.* **14**, 1237426 (2023). 37810401 10.3389/fpls.2023.1237426 PMC10552921

[132] Mokhtar, M. M., Abd-Elhalim, H. M. & El Allali, A. A large-scale assessment of the quality of plant genome assemblies using the LTR assembly index. *AoB Plants* **15**, plad015 (2023). 37197714 10.1093/aobpla/plad015 PMC10184434

[133] Bioinformatics Platform. *UM6P* https://bioinformatics.um6p.ma/platform (Bioinformatics Platform, 2024).

[134] Cock, P. J. A. et al. Biopython: freely available Python tools for computational molecular biology and bioinformatics. *Bioinformatics* **25**, 1422–1423 (2009). 19304878 10.1093/bioinformatics/btp163 PMC2682512

[135] Angermueller, C., Pärnamaa, T., Parts, L. & Stegle, O. Deep learning for computational biology. *Mol. Syst. Biol.* **12**, 878 (2016). 27474269 10.15252/msb.20156651 PMC4965871

[136] Liu, R., Mancuso, C. A., Yannakopoulos, A., Johnson, K. A. & Krishnan, A. Supervised learning is an accurate method for network-based gene classification. *Bioinformatics* **36**, 3457–3465 (2020). 32129827 10.1093/bioinformatics/btaa150 PMC7267831

[137] Alexander, D. H., Novembre, J. & Lange, K. Fast model-based estimation of ancestry in unrelated individuals. *Genome Res.* **19**, 1655–1664 (2009). 19648217 10.1101/gr.094052.109 PMC2752134

[138] Liu, B. et al. Chromosome-level genome assembly of the endangered plant *Tetraena mongolica*. *DNA Res.* **30**, dsad004 (2023). 36999569 10.1093/dnares/dsad004 PMC10113878

[139] Burgin, J. et al. The European Nucleotide Archive in 2022. *Nucleic Acids Res.* **51**, D121–D125 (2023). 36399492 10.1093/nar/gkac1051 PMC9825583

[140] Bolger, A. M., Lohse, M. & Usadel, B. Trimmomatic: a flexible trimmer for Illumina sequence data. *Bioinformatics* **30**, 2114–2120 (2014). 24695404 10.1093/bioinformatics/btu170 PMC4103590

[141] Bankevich, A. et al. SPAdes: a new genome assembly algorithm and its applications to single-cell sequencing. *J. Comput. Biol.* **19**, 455–477 (2012). 22506599 10.1089/cmb.2012.0021 PMC3342519

[142] Gurevich, A., Saveliev, V., Vyahhi, N. & Tesler, G. QUAST: quality assessment tool for genome assemblies. *Bioinformatics* **29**, 1072–1075 (2013). 23422339 10.1093/bioinformatics/btt086 PMC3624806

[143] Goecks, J., Nekrutenko, A. & Taylor, J. & The Galaxy Team. Galaxy: a comprehensive approach for supporting accessible, reproducible, and transparent computational research in the life sciences. *Genome Biol.* **11**, R86 (2010). 20738864 10.1186/gb-2010-11-8-r86 PMC2945788

[144] Katoh, K., Rozewicki, J. & Yamada, K. D. MAFFT online service: multiple sequence alignment, interactive sequence choice and visualization. *Brief. Bioinform.* **20**, 1160–1166 (2019). 28968734 10.1093/bib/bbx108 PMC6781576

[145] Rozas, J. et al. DnaSP 6: dna sequence polymorphism analysis of large data sets. *Mol. Biol. Evol.* **34**, 3299–3302 (2017). 29029172 10.1093/molbev/msx248

[146] Leigh, J. W. & Bryant, D. popart: full-feature software for haplotype network construction. *Methods Ecol. Evol.* **6**, 1110–1116 (2015).

[147] Ogwang, J., Bariche, M. & Bos, A. R. Genetic diversity and phylogenetic relationships of threadfin breams (*Nemipterus* spp.) from the Red Sea and eastern Mediterranean Sea. *Genome* **64**, 207–216 (2021). 32678985 10.1139/gen-2019-0163

[148] Bos, A. R. et al. Anti-Lessepsian migration rectified: the Comber Serranus cabrilla (L. 1758) existed in the Red Sea prior to the Suez Canal opening. *Mar. Biol.* **167**, 126 (2020).

[149] Whitlock, M. C. & McCauley, D. E. Indirect measures of gene flow and migration: FST≠1/(4Nm+1). *Heredity* **82**, 117–125 (1999). 10098262 10.1038/sj.hdy.6884960

[150] Li, H. & Durbin, R. Fast and accurate long-read alignment with Burrows–Wheeler transform. *Bioinformatics* **26**, 589–595 (2010). 20080505 10.1093/bioinformatics/btp698 PMC2828108

[151] Li, H. et al. The Sequence Alignment/Map format and SAMtools. *Bioinformatics* **25**, 2078–2079 (2009). 19505943 10.1093/bioinformatics/btp352 PMC2723002

[152] Danecek, P. et al. Twelve years of SAMtools and BCFtools. *GigaScience* **10**, giab008 (2021). 33590861 10.1093/gigascience/giab008 PMC7931819

[153] Robinson, J. T. et al. Integrative genomics viewer. *Nat. Biotechnol.* **29**, 24–26 (2011). 21221095 10.1038/nbt.1754 PMC3346182

[154] Mughal, M. R. & DeGiorgio, M. Properties and unbiased estimation of *F*- and *D*-statistics in samples containing related and inbred individuals. *Genetics* **220**, iyab090 (2022). 34849832 10.1093/genetics/iyab090 PMC8733448

[155] Purcell, S. et al. PLINK: a tool set for whole-genome association and population-based linkage analyses. *Am. J. Hum. Genet.* **81**, 559–575 (2007). 17701901 10.1086/519795 PMC1950838

[156] Garcia-Erill, G. & Albrechtsen, A. Evaluation of model fit of inferred admixture proportions. *Mol. Eco. Resour.* **20**, 936–949 (2020). 10.1111/1755-0998.13171 32323416

[157] Masoodi, K. Z., Lone, S. M. & Rasool, R. S. Genomic DNA extraction from the plant leaves using the CTAB method. In *Advanced Methods in Molecular Biology and Biotechnology* (eds. Masoodi, K. Z., Lone, S. M. & Rasool, R. S.) 37–44 10.1016/B978-0-12-824449-4.00007-4 (Academic Press, 2021).

[158] Yoshikawa, M. Exorphin-opioid active peptides of exogenous origin. In *Handbook of Biologically Active Peptides* (ed Kastin A. J.) 1365–1371 10.1016/B978-012369442-3/50193-8 (Academic Press, 2006).

[159] Makoni, M. South Africa launches a central biobank network. *Nat Africa*10.1038/D44148-023-00078-3 (2023).

[160] Manheim, B. S. Regulation of synthetic biology under the Nagoya Protocol. *Nat Biotechnol* **34**, 1104–1105 (2016). 27824851 10.1038/nbt.3716

[161] Convention on Biological Diversity. *Cartagena Protocol on Biosafety to the Convention on Biological Diversity* (2000) (Convention on Biological Diversity, 2025).

[162] Wynberg, R. & Chennells, R. Green Diamonds of the South: an overview of the San-Hoodia Case. In *Indigenous Peoples, Consent and Benefit Sharing* (eds. Wynberg, R., Schroeder, D. & Chennells, R.) 89–124 (Springer Netherlands, Dordrecht, 2009).

[163] Elguellab, A. & Ezzahid, E. *Production Networks, Sectoral Shocks And Aggregate Volatility in a Developing Economy: Insights from Morocco* https://www.policycenter.ma/sites/default/files/2023-11/RP_05-23%20%28Ali%20Elguellab%20%26%20Elhadj%20Ezzahid%29.pdf (2023).

