# Supplementary material for: Unlocking the African bioeconomy and strengthening biodiversity conservation through genomics and bioinformatics
Source: NPJ Biodivers. 2025 Jul 29;4:29. doi: 10.1038/s44185-025-00102-9 (PMC12307939; doi:10.1038/s44185-025-00102-9)
Supplement: Supplementary file 1 — Supplementary Information [file 44185_2025_102_MOESM1_ESM.pdf]

Supplementary information

Figure S1: The AfricaBP Open Institute 2024 regional workshops are organized in a model previously described in Sharaf, et al., 2024, using local resources.

Figure S2: AfricaBP Open Institute 2024 regional workshops.

Figure S3: AfricaBP Open Institute 2024 regional workshops reveal an overlap of attendance.

Figure S4: The AfricaBP Open Institute 2024 regional workshops built biodiversity genomics and bioinformatics awareness for an estimated 75% of non-AfricaBP members obtained from pre-workshop survey analysis.

Figure S5: Nearly 50% of post-workshop survey respondents were satisfied with the workshop and the majority expressed an interest in similar workshops to be organised in the future.

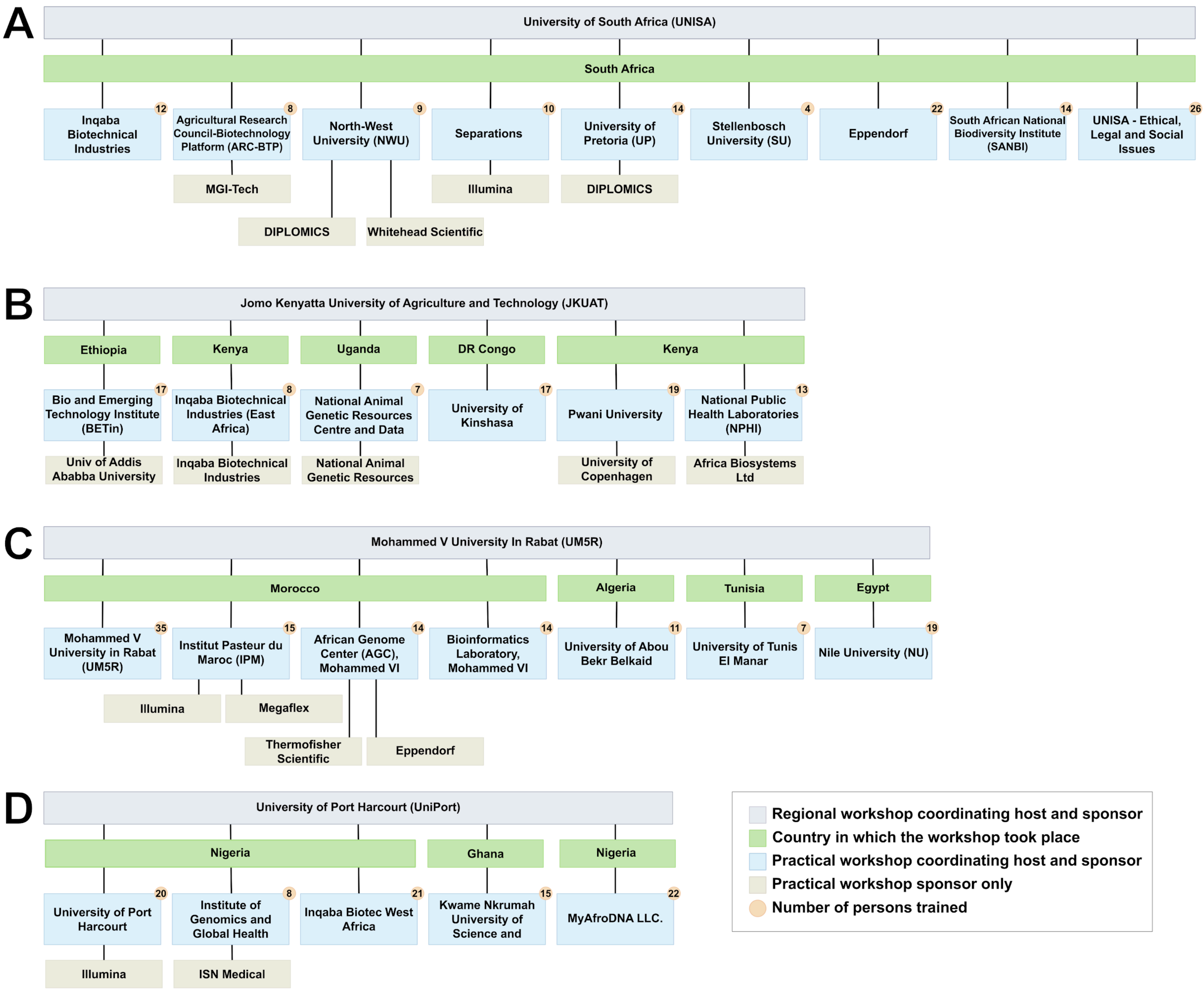
A
University of South Africa (UNISA)
South Africa
Inqaba Biotechnical Industries 12
Agricultural Research Council-Biotechnology Platform (ARC-BTP) 8
North-West University (NWU) 9
Separations 10
University of Pretoria (UP) 14
Stellenbosch University (SU) 4
Eppendorf 22
South African National Biodiversity Institute (SANBI) 14
UNISA - Ethical, Legal and Social Issues 26
MGI-Tech
Illumina
DIPLOMICS
DIPLOMICS
Whitehead Scientific
B
Jomo Kenyatta University of Agriculture and Technology (JKUAT)
Ethiopia
Kenya
Uganda
DR Congo
Kenya
Bio and Emerging Technology Institute (BETin) 17
Inqaba Biotechnical Industries (East Africa) 8
National Animal Genetic Resources Centre and Data 7
University of Kinshasa 17
Pwani University 19
National Public Health Laboratories (NPHI) 13
Univ of Addis Ababba University
Inqaba Biotechnical Industries
National Animal Genetic Resources
University of Copenhagen
Africa Biosystems Ltd
C
Mohammed V University In Rabat (UM5R)
Morocco
Algeria
Tunisia
Egypt
Mohammed V University in Rabat (UM5R) 35
Institut Pasteur du Maroc (IPM) 15
African Genome Center (AGC), Mohammed VI 14
Bioinformatics Laboratory, Mohammed VI 14
University of Abou Bekr Belkaid 11
University of Tunis El Manar 7
Nile University (NU) 19
Illumina
Megaflex
Thermofisher Scientific
Eppendorf
D
University of Port Harcourt (UniPort)
Nigeria
Ghana
Nigeria
University of Port Harcourt 20
Institute of Genomics and Global Health 8
Inqaba Biotec West Africa 21
Kwame Nkrumah University of Science and 15
MyAfroDNA LLC. 22
Illumina
ISN Medical
Regional workshop coordinating host and sponsor
Country in which the workshop took place
Practical workshop coordinating host and sponsor
Practical workshop sponsor only
Number of persons trained

Figure S1: The AfricaBP Open Institute 2024 regional workshops are organized in a model previously described in Sharaf, et al., 2024, using local resources. A: Hub and spoke model for the delivery of the AfricaBP Open Institute Southern Africa regional workshop 2024. **B:** Hub and spoke model for the delivery of the AfricaBP Open Institute East and Central Africa regional workshop 2024. **C:** Hub and spoke model for the delivery of the AfricaBP Open Institute Northern Africa regional workshop 2024. **D:** Hub and spoke model for the delivery of the AfricaBP Open Institute West Africa regional workshop 2024. The hub includes the coordinating host institutions (highlighted in grey) while the spoke includes the various practical workshops at different satellite locations (highlighted in blue) across several African countries (highlighted in green) and their respective sponsors (highlighted in brown). The numbers (highlighted in orange) depict the number of persons trained by the practical host institution during the 2024 series of the AfricaBP Open Institute workshops.

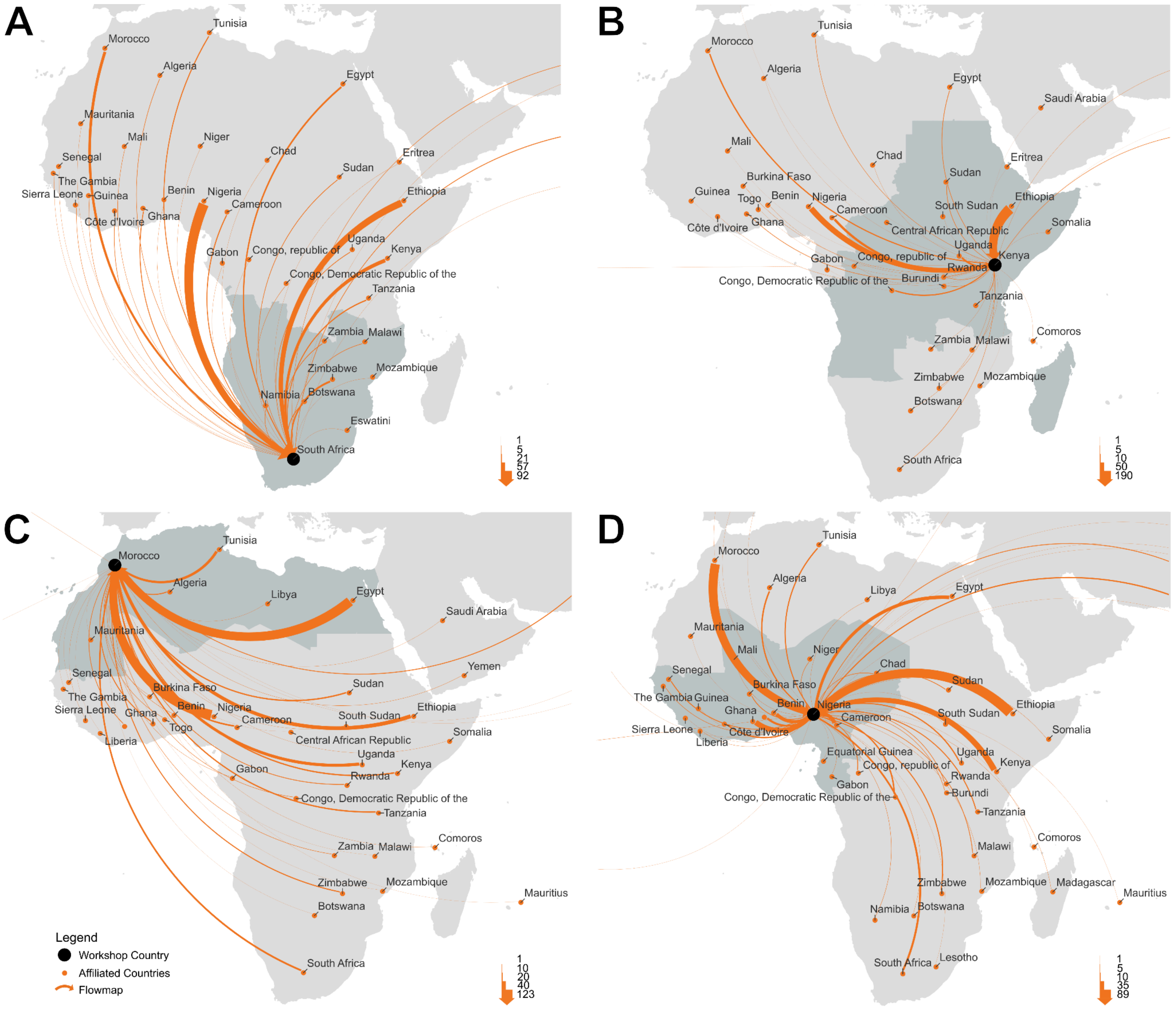
A
B
C
D
Legend
Workshop Country
Affiliated Countries
Flowmap
1
5
21
57
92
1
5
10
50
190
1
10
20
40
123
1
5
10
35
89

Figure S2: AfricaBP Open Institute 2024 regional workshops. Map of Africa showing countries of origin of registered participants for each of the AfricaBP Open Institute regional workshops held in 2024. **A:** Diagram showing countries of origin of registered participants during the Southern Africa regional workshop. **B:** Diagram showing countries of origin of registered participants during the Eastern and Central Africa regional workshop. **C:** Diagram showing countries of origin of registered participants during the Northern Africa regional workshop. **D:** Diagram showing countries of origin of registered participants during the Western Africa regional workshop (see Sharaf, et al., 2024 for model[4]). This map was generated with AfricaBP registration information using QGIS software[161], courtesy of ArcWorld Supplement.

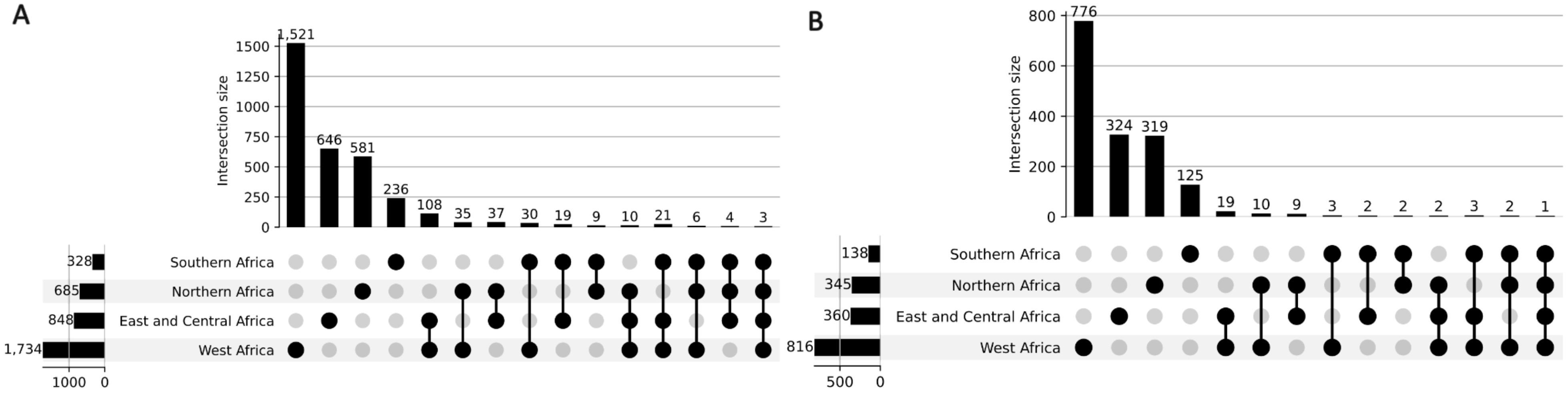
A
Intersection size
1,521
646
581
236
108
35
37
30
19
9
10
21
6
4
3
Southern Africa
Northern Africa
East and Central Africa
West Africa
328
685
848
1,734
B
776
324
319
125
19
10
9
3
2
2
2
3
2
1
138
345
360
816

Figure S3: AfricaBP Open Institute 2024 regional workshops reveal overlapping attendance. The diagrams show UpSetPlots for registered participants, and confirmed participants. **A:** Registered participants. This diagram shows the number of registered participants. The AfricaBP Open Institute regional workshop in Northern Africa had 685 participants; 581 of these did not register to attend any of the AfricaBP Open Institute regional workshops in East and Central Africa, Southern Africa, and West Africa. Only 9 participants attended both the North and Southern Africa regional workshops. **B:** Snapshot headcount of participants. This diagram shows the snapshot headcount during the awareness-building session of all the workshops in 2024. This headcount was conducted using Zoom login IDs for virtual participants and physical attendance counts. It does not include counts of participants who participated via YouTube and Twitter which has thousands of views across AfricaBP YouTube and Twitter platforms, as at the time of writing this paper. We also had instances where several registered participants preferred to attend the awareness-building session of a regional workshop via their university's conference room using a single Zoom ID in which case the headcount was 1, even though there were several participants (see Sharaf, et al., 2024 for details[4]). This Figure was generated by analyzing the Participants' ID information with the UpSetPlot Python package[162].

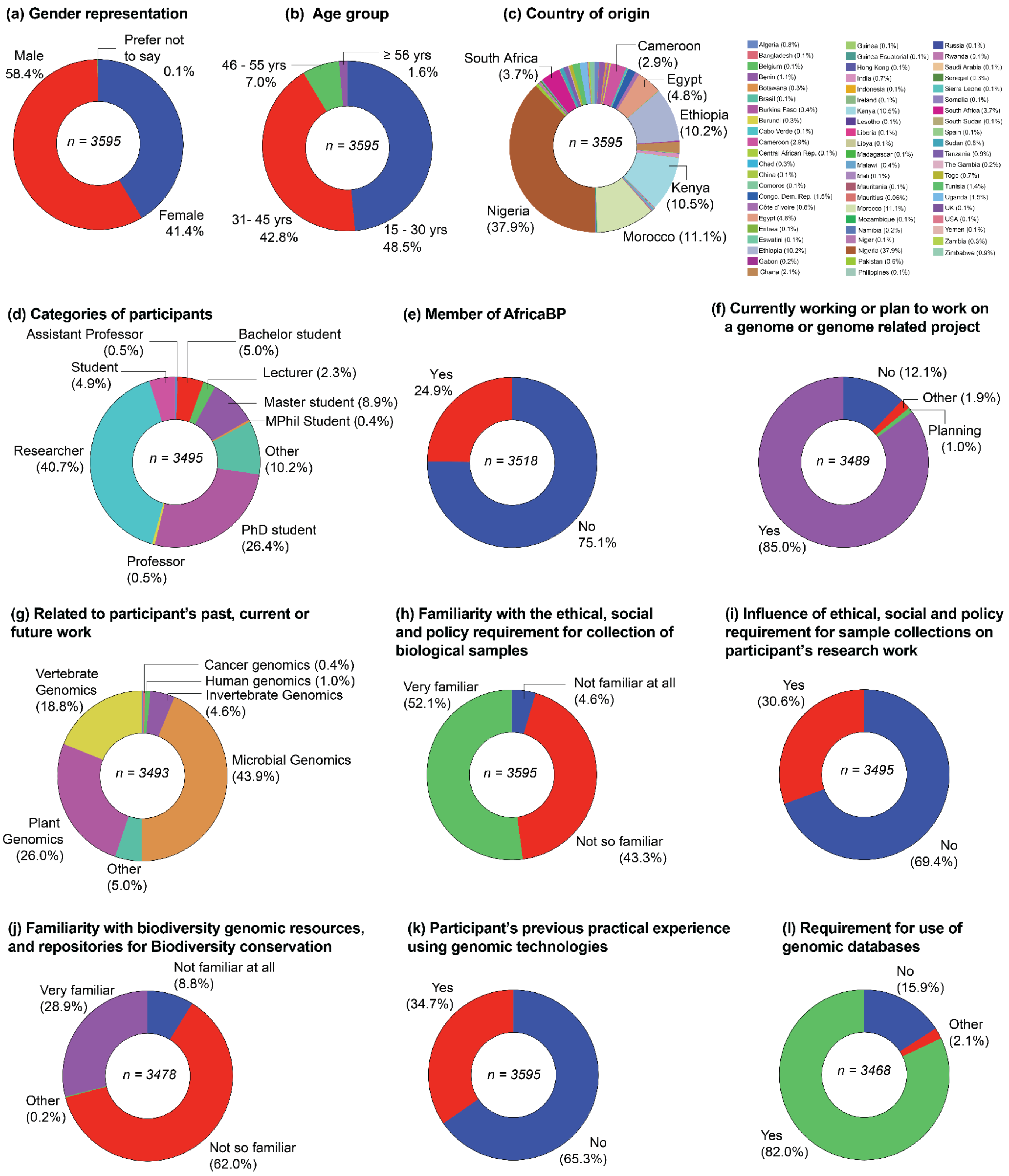

(a) Gender representation
Male 58.4%
Prefer not to say 0.1%
n = 3595
Female 41.4%
(b) Age group
46 - 55 yrs 7.0%
≥ 56 yrs 1.6%
n = 3595
31- 45 yrs 42.8%
15 - 30 yrs 48.5%
(c) Country of origin
South Africa (3.7%)
Cameroon (2.9%)
Egypt (4.8%)
Ethiopia (10.2%)
n = 3595
Kenya (10.5%)
Nigeria (37.9%)
Morocco (11.1%)
Algeria (0.8%)
Bangladesh (0.1%)
Belgium (0.1%)
Benin (1.1%)
Botswana (0.3%)
Brasil (0.1%)
Burkina Faso (0.4%)
Burundi (0.3%)
Cabo Verde (0.1%)
Cameroon (2.9%)
Central African Rep. (0.1%)
Chad (0.3%)
China (0.1%)
Comoros (0.1%)
Congo, Dem. Rep. (1.5%)
Côte d'Ivoire (0.8%)
Egypt (4.8%)
Eritrea (0.1%)
Eswatini (0.1%)
Ethiopia (10.2%)
Gabon (0.2%)
Ghana (2.1%)
Guinea (0.1%)
Guinea Ecuatorial (0.1%)
Hong Kong (0.1%)
India (0.7%)
Indonesia (0.1%)
Ireland (0.1%)
Kenya (10.5%)
Lesotho (0.1%)
Liberia (0.1%)
Libya (0.1%)
Madagascar (0.1%)
Malawi (0.4%)
Mali (0.1%)
Mauritania (0.1%)
Mauritius (0.06%)
Morocco (11.1%)
Mozambique (0.1%)
Namibia (0.2%)
Niger (0.1%)
Nigeria (37.9%)
Pakistan (0.6%)
Philippines (0.1%)
Russia (0.1%)
Rwanda (0.4%)
Saudi Arabia (0.1%)
Senegal (0.3%)
Sierra Leone (0.1%)
Somalia (0.1%)
South Africa (3.7%)
South Sudan (0.1%)
Spain (0.1%)
Sudan (0.8%)
Tanzania (0.9%)
The Gambia (0.2%)
Togo (0.7%)
Tunisia (1.4%)
Uganda (1.5%)
UK (0.1%)
USA (0.1%)
Yemen (0.1%)
Zambia (0.3%)
Zimbabwe (0.9%)
(d) Categories of participants
Assistant Professor (0.5%)
Bachelor student (5.0%)
Student (4.9%)
Lecturer (2.3%)
Master student (8.9%)
MPhil Student (0.4%)
Researcher (40.7%)
n = 3495
Other (10.2%)
PhD student (26.4%)
Professor (0.5%)
(e) Member of AfricaBP
Yes 24.9%
n = 3518
No 75.1%
(f) Currently working or plan to work on a genome or genome related project
No (12.1%)
Other (1.9%)
Planning (1.0%)
n = 3489
Yes (85.0%)
(g) Related to participant's past, current or future work
Vertebrate Genomics (18.8%)
Cancer genomics (0.4%)
Human genomics (1.0%)
Invertebrate Genomics (4.6%)
n = 3493
Microbial Genomics (43.9%)
Plant Genomics (26.0%)
Other (5.0%)
(h) Familiarity with the ethical, social and policy requirement for collection of biological samples
Very familiar (52.1%)
Not familiar at all (4.6%)
n = 3595
Not so familiar (43.3%)
(i) Influence of ethical, social and policy requirement for sample collections on participant's research work
Yes (30.6%)
n = 3495
No (69.4%)
(j) Familiarity with biodiversity genomic resources, and repositories for Biodiversity conservation
Not familiar at all (8.8%)
Very familiar (28.9%)
n = 3478
Other (0.2%)
Not so familiar (62.0%)
(k) Participant's previous practical experience using genomic technologies
Yes (34.7%)
n = 3595
No (65.3%)
(l) Requirement for use of genomic databases
No (15.9%)
Other (2.1%)
n = 3468
Yes (82.0%)

(m) Impact of ethical, social and policy requirement for sample collections on participant's research work (*n = 791)*

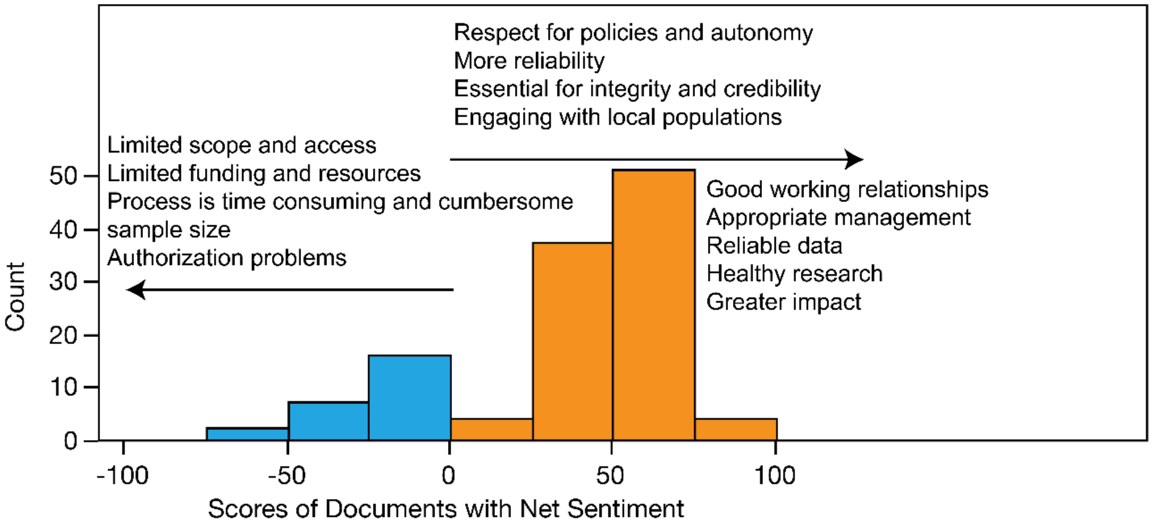

(n) Topics of interest for participants in getting training during the workshop (*n = 2818)*

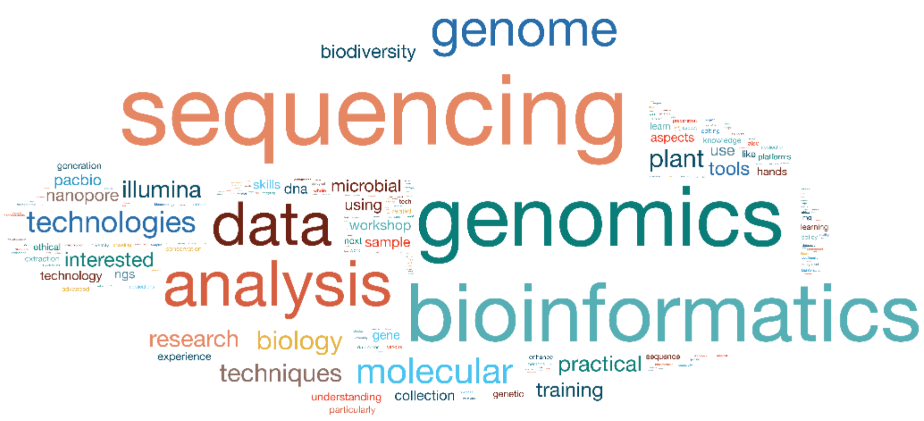

(p) Participants feeling on knowledge gained in this workshop and how this will impact their institution in Africa afterwards *(n = 3526)*

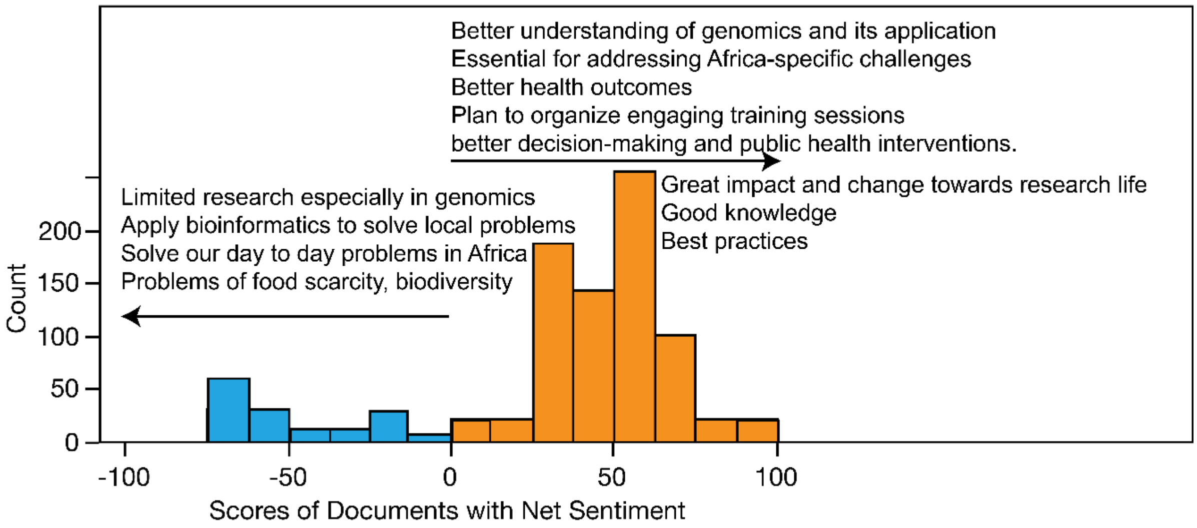

Figure S4: The AfricaBP Open Institute 2024 regional workshops built biodiversity genomics and bioinformatics awareness for an estimated 75% of non-AfricaBP members obtained from pre-workshop survey analysis. (a-e) General statistics on participants' gender, age, and country of origin. (f-i) 85% of participants are currently working on genome projects with nearly half being very familiar with ethical, social, and policy requirements for sample collection. (j) Surprisingly, 62% are not so familiar with biodiversity resources and repositories for conservation. (k-l) Participants had moderate experience in genomic technologies (35%) and 82% agreed with their need for genomic databases. (m) Sentiment analysis from 791 responses reveals a largely positive attitude towards ethical, social, and policy requirements for sample collection promotes respect, research reliability, engages local communities, and eventually leads to greater impact. Some encountered problems are associated with permits and sample size and limited funding and access to resources. (n) Cloud analysis showed that participants were keen to undertake 'data analysis', learned 'bioinformatics techniques' and 'sequencing' approaches. (p) Sentiment analysis from 3526 responses reveals a largely positive attitude on how the knowledge gained will help their institutions across Africa afterward, namely with better health outcomes, ways to address Africa-specific challenges, and also establish best practices. Statistical and sentiment analysis were conducted using JMP (Version *17.0*. SAS Institute Inc., Cary, NC, 1989-2023)[163].

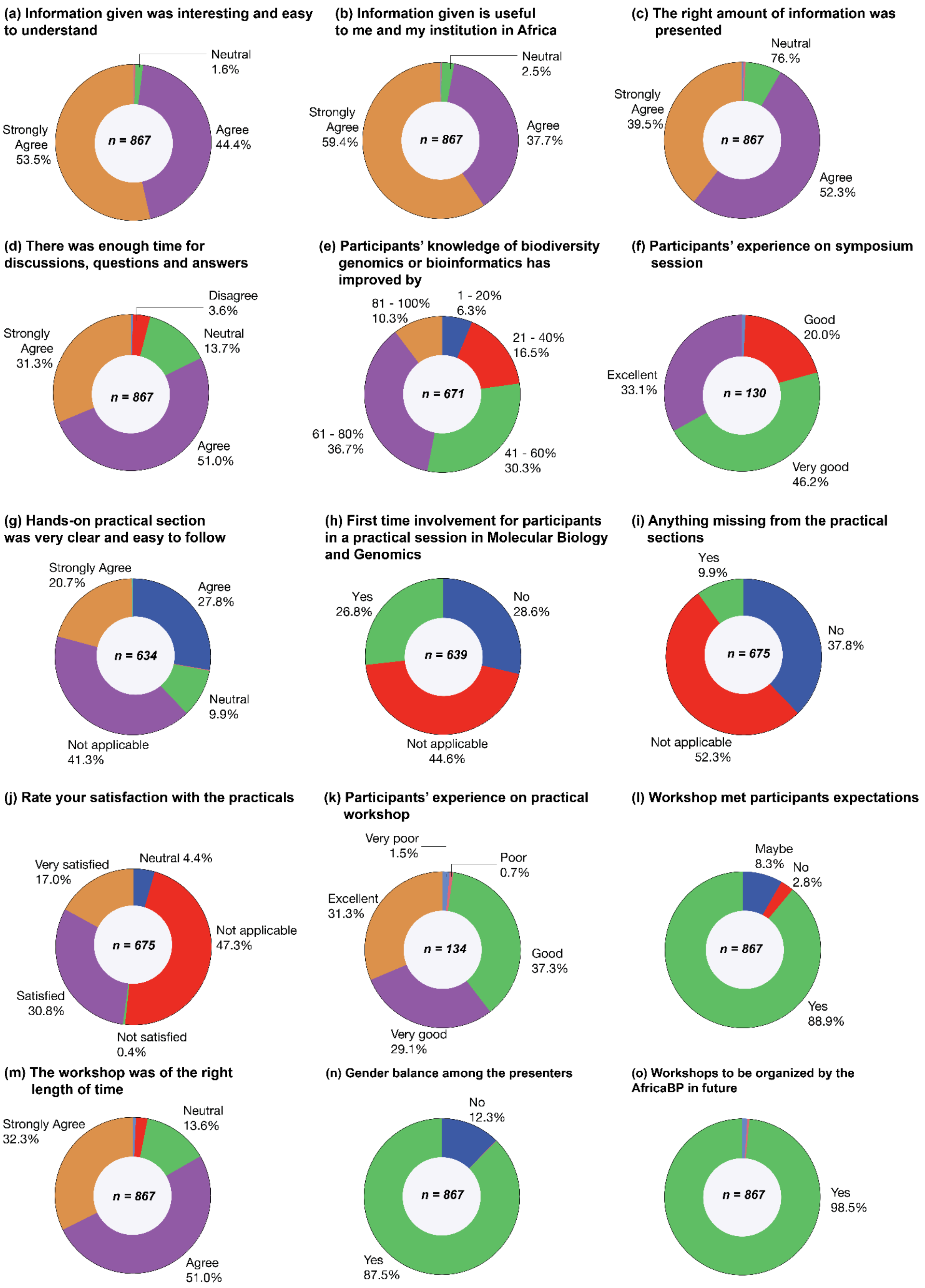
(a) Information given was interesting and easy to understand
Neutral 1.6%
Strongly Agree 53.5%
n = 867
Agree 44.4%
(b) Information given is useful to me and my institution in Africa
Neutral 2.5%
Strongly Agree 59.4%
n = 867
Agree 37.7%
(c) The right amount of information was presented
Neutral 76.%
Strongly Agree 39.5%
n = 867
Agree 52.3%
(d) There was enough time for discussions, questions and answers
Disagree 3.6%
Strongly Agree 31.3%
Neutral 13.7%
n = 867
Agree 51.0%
(e) Participants' knowledge of biodiversity genomics or bioinformatics has improved by
81 - 100% 10.3%
1 - 20% 6.3%
21 - 40% 16.5%
n = 671
61 - 80% 36.7%
41 - 60% 30.3%
(f) Participants' experience on symposium session
Good 20.0%
Excellent 33.1%
n = 130
Very good 46.2%
(g) Hands-on practical section was very clear and easy to follow
Strongly Agree 20.7%
Agree 27.8%
n = 634
Neutral 9.9%
Not applicable 41.3%
(h) First time involvement for participants in a practical session in Molecular Biology and Genomics
Yes 26.8%
No 28.6%
n = 639
Not applicable 44.6%
(i) Anything missing from the practical sections
Yes 9.9%
No 37.8%
n = 675
Not applicable 52.3%
(j) Rate your satisfaction with the practicals
Very satisfied 17.0%
Neutral 4.4%
Not applicable 47.3%
n = 675
Satisfied 30.8%
Not satisfied 0.4%
(k) Participants' experience on practical workshop
Very poor 1.5%
Poor 0.7%
Excellent 31.3%
n = 134
Good 37.3%
Very good 29.1%
(l) Workshop met participants expectations
Maybe 8.3%
No 2.8%
n = 867
Yes 88.9%
(m) The workshop was of the right length of time
Neutral 13.6%
Strongly Agree 32.3%
n = 867
Agree 51.0%
(n) Gender balance among the presenters
No 12.3%
n = 867
Yes 87.5%
(o) Workshops to be organized by the AfricaBP in future
n = 867
Yes 98.5%

(p) Topics missing or issues from the lecture session (*n* =160)

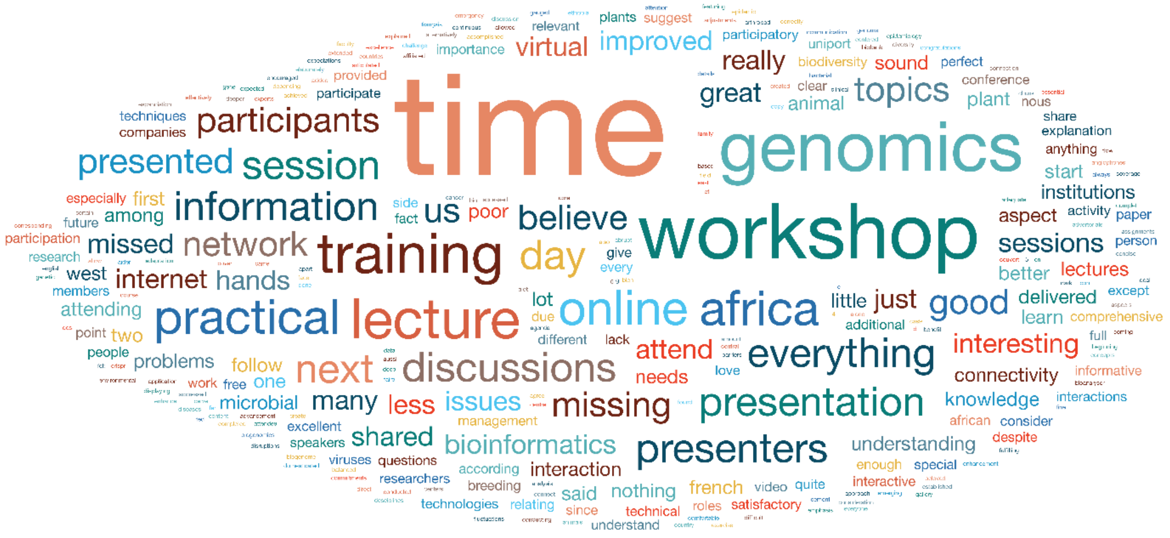

(q) Topics missing or issues from the practical session (*n* = 69)

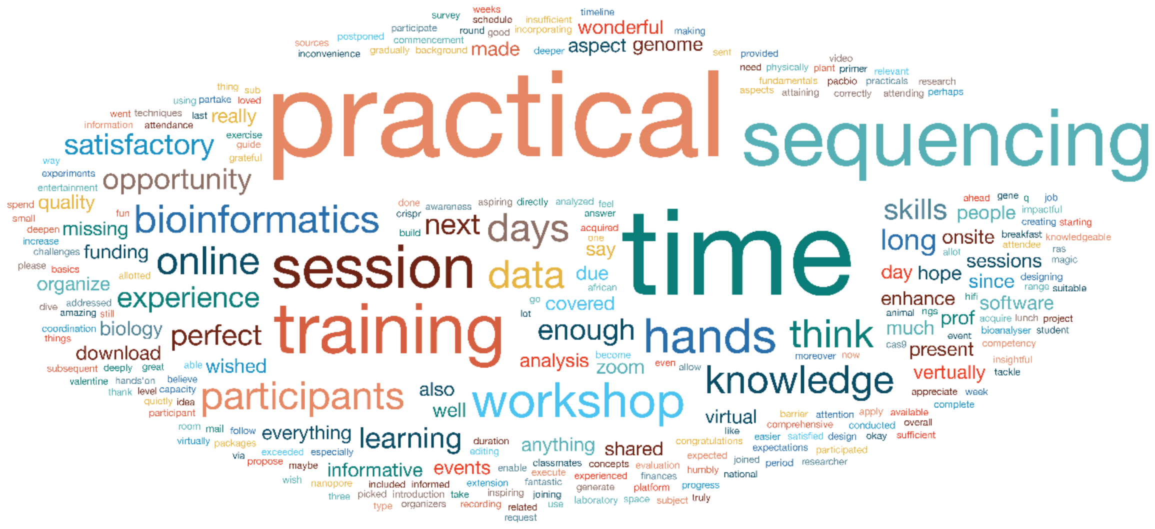

(r) Topics that was not covered in the workshop participants expected (*n* = 478)

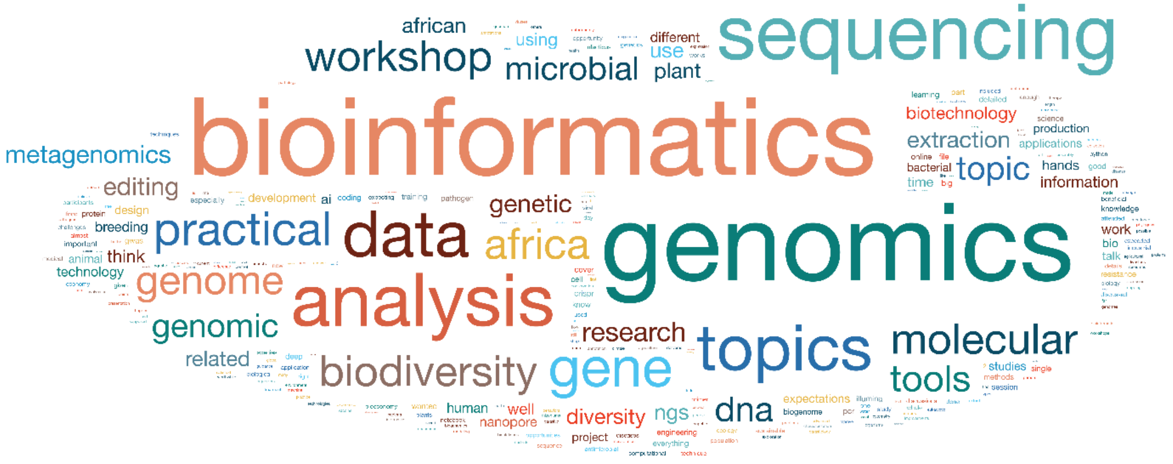

(s) Participants queries with workshop (*n* = *152*)

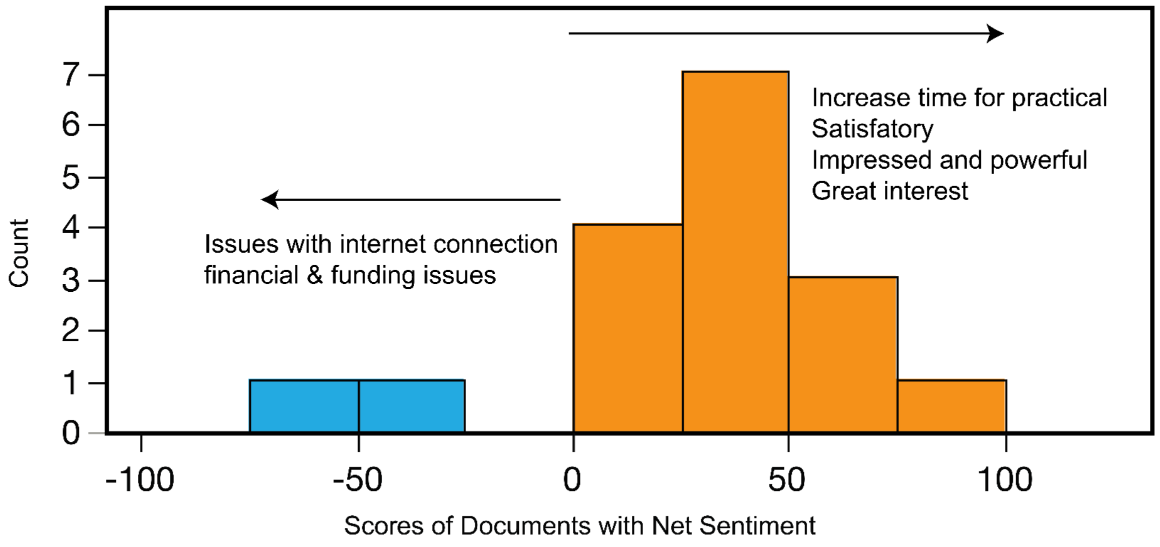

(t) Participants feedback on workshop (*n* = *181*)

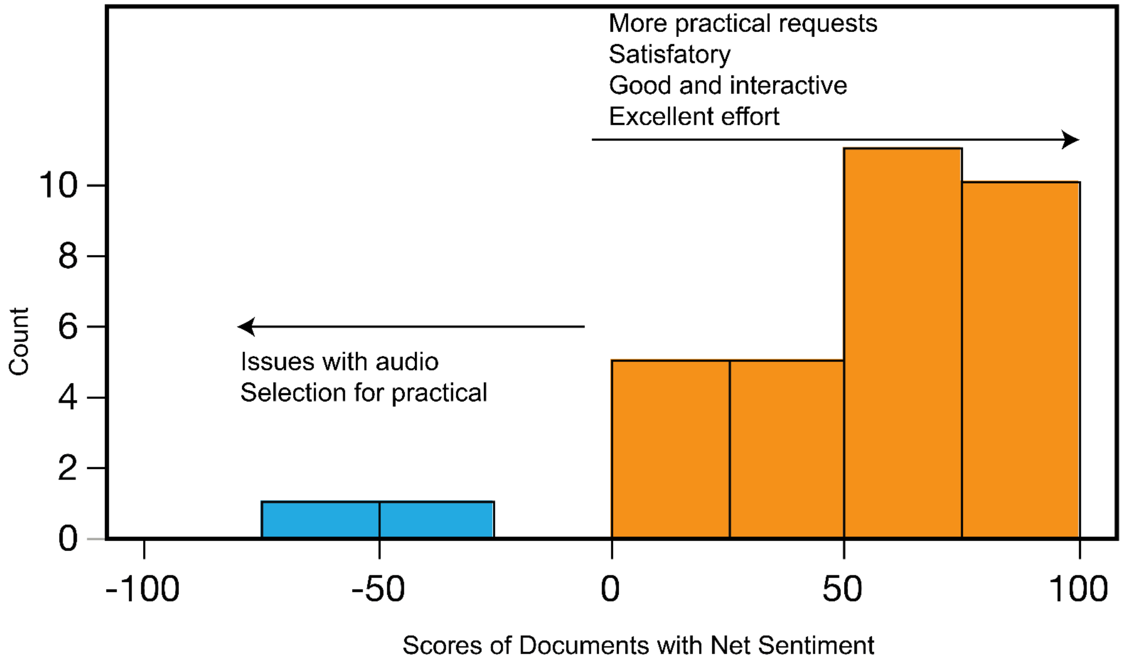

Figure S5: Nearly 50% of post-workshop survey respondents were satisfied with the workshop and the majority expressed an interest in similar workshops to be organised in the future. (a-d) At least 40% of respondents strongly agreed that the information given was interesting, would be helpful to them, and was of the right

amount. (e) More than 75% confirmed that their knowledge of biodiversity genomics improved by at least 40%. (f) At least 20% had a good symposium experience. (g-i) There was strong agreement on the ease of the practical session (at least 55%). (j) Nearly 50% expressed their satisfaction with the practical sessions and found their experience to be good (at least 37%). (l-m) Nearly 90% felt the workshop met their expectations and the workshop was of the right length of time. (n-o) Nearly 90% agreed for similar workshops to be organized in the future. (p-r) Word cloud analysis from respondents who attended the lecture and practical session identified ‘time’ as a major issue along with poor ‘network’. (s) Sentiment analysis showed overall positive responses to queries they raised, and they expressed satisfaction with the workshop as an ‘excellent effort’ with the need for more practical requests. Statistical and sentiment analysis was conducted using JMP (Version *17.0*. SAS Institute Inc., Cary, NC, 1989-2023)[163].
